# Cumulative Roles for Epstein-Barr Virus, Human Endogenous Retroviruses, and Human Herpes Virus-6 in Driving an Inflammatory Cascade Underlying MS Pathogenesis

**DOI:** 10.3389/fimmu.2021.757302

**Published:** 2021-11-01

**Authors:** Ute-Christiane Meier, Richard Christopher Cipian, Abbas Karimi, Ranjan Ramasamy, Jaap Michiel Middeldorp

**Affiliations:** ^1^ Institut für Laboratoriumsmedizin, Klinikum der Universität München, München, Germany; ^2^ Department of Psychological Medicine, Institute of Psychiatry, Psychology and Neuroscience, King’s College London, London, United Kingdom; ^3^ Department of Biology, Crafton Hills College, Yucaipa, CA, United States; ^4^ Department of Molecular Medicine, Faculty of Advanced Medical Sciences, Tabriz University of Medical Sciences, Tabriz, Iran; ^5^ ID-FISH Technology Inc., Miltipas, CA, United States; ^6^ Department of Pathology, Amsterdam University Medical Center, VUMC, Amsterdam, Netherlands

**Keywords:** Epstein-Barr virus, human endogenous retrovirus-W, inflammatory cascade, molecular mimicry, multiple sclerosis, human herpesvirus-6

## Abstract

Roles for viral infections and aberrant immune responses in driving localized neuroinflammation and neurodegeneration in multiple sclerosis (MS) are the focus of intense research. Epstein-Barr virus (EBV), as a persistent and frequently reactivating virus with major immunogenic influences and a near 100% epidemiological association with MS, is considered to play a leading role in MS pathogenesis, triggering localized inflammation near or within the central nervous system (CNS). This triggering may occur directly *via* viral products (RNA and protein) and/or indirectly *via* antigenic mimicry involving B-cells, T-cells and cytokine-activated astrocytes and microglia cells damaging the myelin sheath of neurons. The genetic MS-risk factor HLA-DR2b (DRB1*1501β, DRA1*0101α) may contribute to aberrant EBV antigen-presentation and anti-EBV reactivity but also to mimicry-induced autoimmune responses characteristic of MS. A central role is proposed for inflammatory EBER1, EBV-miRNA and LMP1 containing exosomes secreted by viable reactivating EBV+ B-cells and repetitive release of EBNA1-DNA complexes from apoptotic EBV+ B-cells, forming reactive immune complexes with EBNA1-IgG and complement. This may be accompanied by cytokine- or EBV-induced expression of human endogenous retrovirus-W/-K (HERV-W/-K) elements and possibly by activation of human herpesvirus-6A (HHV-6A) in early-stage CNS lesions, each contributing to an inflammatory cascade causing the relapsing-remitting neuro-inflammatory and/or progressive features characteristic of MS. Elimination of EBV-carrying B-cells by antibody- and EBV-specific T-cell therapy may hold the promise of reducing EBV activity in the CNS, thereby limiting CNS inflammation, MS symptoms and possibly reversing disease. Other approaches targeting HHV-6 and HERV-W and limiting inflammatory kinase-signaling to treat MS are also being tested with promising results. This article presents an overview of the evidence that EBV, HHV-6, and HERV-W may have a pathogenic role in initiating and promoting MS and possible approaches to mitigate development of the disease.

## Introduction

Multiple sclerosis (MS) is a debilitating neurological condition with a strong autoimmune component and a significant cause of neurological impairment in young adults. MS is characterized by episodic, localized and progressive demyelination as the final result of focal inflammatory lesions, causing progressive or reiterating neuroinflammatory and neurodegenerative changes of the white and gray matter (1). The disease is characterized by immune cell infiltration from the periphery into the central nervous system (CNS), causing localized inflammation and demyelination with axonal damage, leading to autonomic, sensorimotor, and cognitive impairments. The severity and clinical phenotype is dependent on the frequency and distribution of CNS inflammatory lesions as well as the cellular composition and activation status of such lesions (1, 2). Intensive research has focused on autoreactive T- and B-cells as causal mediators, although the trigger for inflammation and autoreactivity remains obscure (1).

The diagnosis of MS is based on neurological examination, magnetic resonance imaging (MRI) and the presence of oligoclonal bands in the cerebrospinal fluid (3, 4). Immunomodulatory therapies are used for the treatment of MS. These disease-modifying treatments (DMTs) reduce the incidence of relapses and impact on progression by dampening the inflammatory signaling and reducing the entry of lymphocytes into the brain (5). The etiology of MS is unknown, but the immune system is thought to be pivotal in the development of MS in genetically predisposed individuals, in addition to environmental risk factors such as smoking, deficiency in sun exposure/vitamin D, and infection (6, 7). There is growing evidence indicating a causal role for viral pathogens in MS, serving as inflammatory agents activating astrocytes and microglia directly or indirectly (8, 9). Several viruses, including Epstein-Barr virus (EBV), Human Herpesvirus-6 (HHV-6), Varicella-zoster virus, John Cunningham virus, and human endogenous retroviruses (HERVs), have been studied in the context of MS. Except for HERVs, these viruses are persistent and cause life-long infections with “cellular stress-triggered” reactivation cycles that may be associated with the relapsing nature of MS (10, 11).

**Figure 1:**
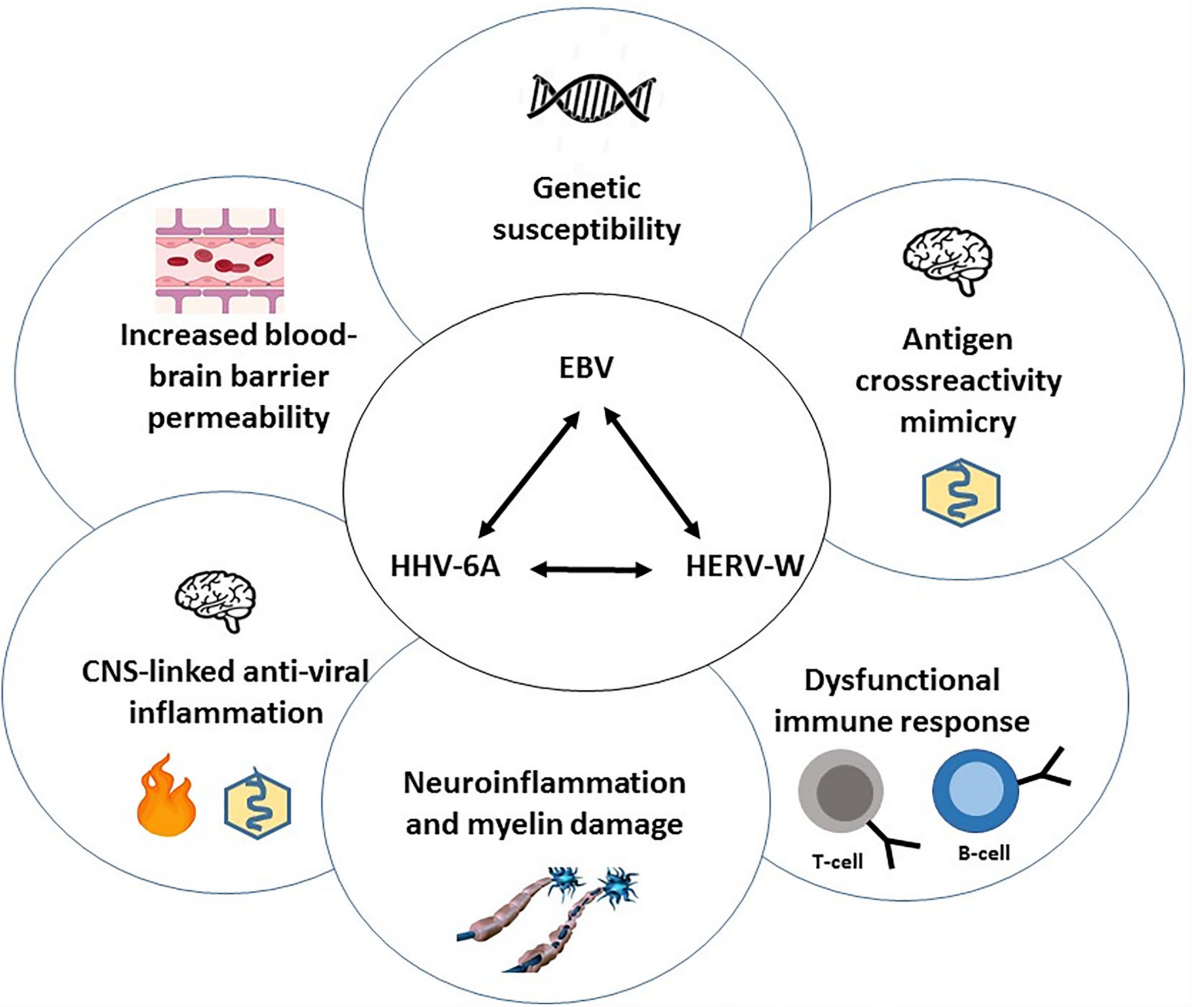
Overview of the central viral cascade causing dysregulation of immune responses, localized CNS-inflammation and neuronal damage underlying MS-pathogenesis. A central role is proposed for Epstein-Barr virus (EBV), a persistent and frequently reactivating virus, that is associated with Multiple Sclerosis (MS) in genetically susceptible individuals (HLA-DRB*1501). Following (“stress”-induced) EBV reactivation in quiescent latency-I EBV-carrying B-cells in lymphoid tissues near the CNS, EBV-encoded gene products, - such as EBER1, miRNA and LMP1 in exosomes secreted by viable reactivating B-cells (latency-II/-III) and EBNA1-DNA complexes released from apoptotic EBV+ B-cells and possibly forming reactive immune complexes with locally produced anti-EBNA1-IgG -, together trigger anti-viral T-/B-/NK-cell-, antibody- and cytokine responses (Type-I IFN, TNF-α, IL-6, -10, -17A) causing localized inflammation. This is associated with spread of viral products, immune complexes, cytokines and the infiltration of (cross-)? reactive lymphocytes *via* a compromised blood-brain barrier causing aberrant activation of CNS-resident microglia and astrocytes and damage to oligodendrocytes (ODCs), leading to CNS inflammatory lesions as characteristic feature of MS. EBV itself and inflammatory cytokines may trigger the expression of endogenous germline-encoded viral sequences (MSRV or HERV-K/-W and HHV6A) in reactive lymphocytes and in inflammatory glia/astrocytes/ODC, which further enhance the localized CNS inflammation. In this inflammatory milieu, shared epitopes between (endogenous) viral and neuronal self-antigens may trigger autoimmune responses in susceptible individuals (HLA-DRB*1501), thus perpetuating CNS inflammation and causing pathogenic microglia activation and neuronal damage in an episodic (CIS), recurrent (RRMS) or progressive (SPMS, PPMS) virus-driven and auto-reactive pathogenic process. Interference with virus-driven inflammatory signaling, reconstitution of a quiescent immunological balance, remyelination and repair of damaged neurons are therefore key to future treatment and curative approaches in MS.

Here we present a hypothesis and testable model (**Figure 1**) suggesting a leading role of EBV infected B-cells in triggering the clinical MS phenotypes in genetically susceptible individuals (i.c. HLA-DR2b) by causing direct focal inflammation near and subsequently within the CNS, inducing reactivation of endogenous viruses (HERVs and HHV6A) and mimicry-driven autoimmunity within the CNS. This inflammatory cascade, overall and in time, leads to deranged (self- and virus-reactive) immune responses with reiterating and/or progressive inflammatory signaling in CNS-resident lymphocytes, glia-cells and astrocytes, associating with destruction of myelin producing oligodendrocytes (ODCs), together causing damage to the protective myelin sheath of neurons leading to axonal damage and progressive neurological disability (2, 9).

## Epstein-Barr Virus and Multiple Sclerosis

### A Brief Description of EBV Biology and Life Cycle

EBV is a γ1 human herpesvirus (HHV4, lymphocryptovirus) that persistently infects up to 95 percent of humans worldwide mainly during childhood. Primary infection in young adults is often symptomatic and referred to as glandular fever or infectious mononucleosis (IM). EBV transforms and immortalizes B-lymphocytes during initial infection as an essential part of its life cycle, but persists as a quiescent (latent) virus in a small number of circulating resting memory B-cells and is thought to infect epithelial cells for virus replication and spread (12–14). Importantly, the bulk of EBV-carrying B-cells appear to home to lymphoid tissues in the head and neck region, proximal to the CNS (15). Dysregulated EBV-infection is associated with several pathologies, including neurological, hematological and autoimmune diseases, e.g. chronic-active EBV infection, MS, Rheumatoid Arthritis (RA) and Systemic Lupus Erythematosus (SLE), as well as multiple cancers, including distinct types of lymphoma and carcinoma (16–20).

EBV is a double-stranded DNA virus encoding approximately 100 open reading frames, which are expressed in tightly regulated latent (transforming and persistent) and replicative (reproductive) gene clusters (21–23). Genes expressed during latency encode proteins like EBV-nuclear-antigen-1 (EBNA-1), essential for viral genome maintenance in dividing cells, linking the viral genome to host cell chromosomes and affecting host gene-expression and the latent membrane proteins-1 and -2 (LMP-1, -2), crucial for inducing and maintaining an immortalized transformed state (default program of latent gene expression, *Latency-II*). During initial B-cell transformation and immortalization, additional EBV nuclear proteins (EBNA-2 to EBNA-6) are expressed (transformation program, *Latency-III*), but their activity is quickly limited by gene silencing *via* CpG-promotor methylation within- and strong T-cell control against Latency-II/-III activated EBV^+^ B-cells (14, 24). The Latency-II and -III stages are only sporadically detectable in lymphoid follicles, where EBV-infected B-cells can proliferate and mimic a germinal center activation program (25). In healthy EBV carriers EBV persists for life in a small number of quiescent circulating memory B-lymphocytes, expressing only EBNA-1 protein when these cells divide (True Latency program, *Latency-I*) (26). Upon cognate antigen encounter within lymphoid tissues, these memory B-cells can switch to IgG-producing plasma cells (13). The molecular switch of memory B-cell to Ig-producing plasma cell activates a three stage EBV lytic-cycle with ultimate virion release and apoptotic cell death. Induction of the lytic phase requires initial triggering of immediate-early viral gene expression encoding highly immunogenic transcription factors Zta and Rta (step-1), followed by early genes encoding enzymes for nucleotide metabolism and viral DNA replication (step-2) and late genes encoding proteins involved in virion assembly (step-3) (27). Virus-encoded small nuclear and nucleolar RNAs and over 40 microRNAs encoded in two gene clusters are abundantly expressed during latent and lytic stages and modulate host gene expression and inflammatory responses, associating with distinct EBV-driven diseases (28–31). Host immune surveillance against EBV is constantly active at a high level (~1% of all T-cells are EBV-antigen responsive) and predominantly directed at latency-II/-III and immediate early gene products, preventing latent B-cell proliferation and lytic activation of EBV (24).

### Humoral Immune Responses Against EBV in MS

**Figure 2:**
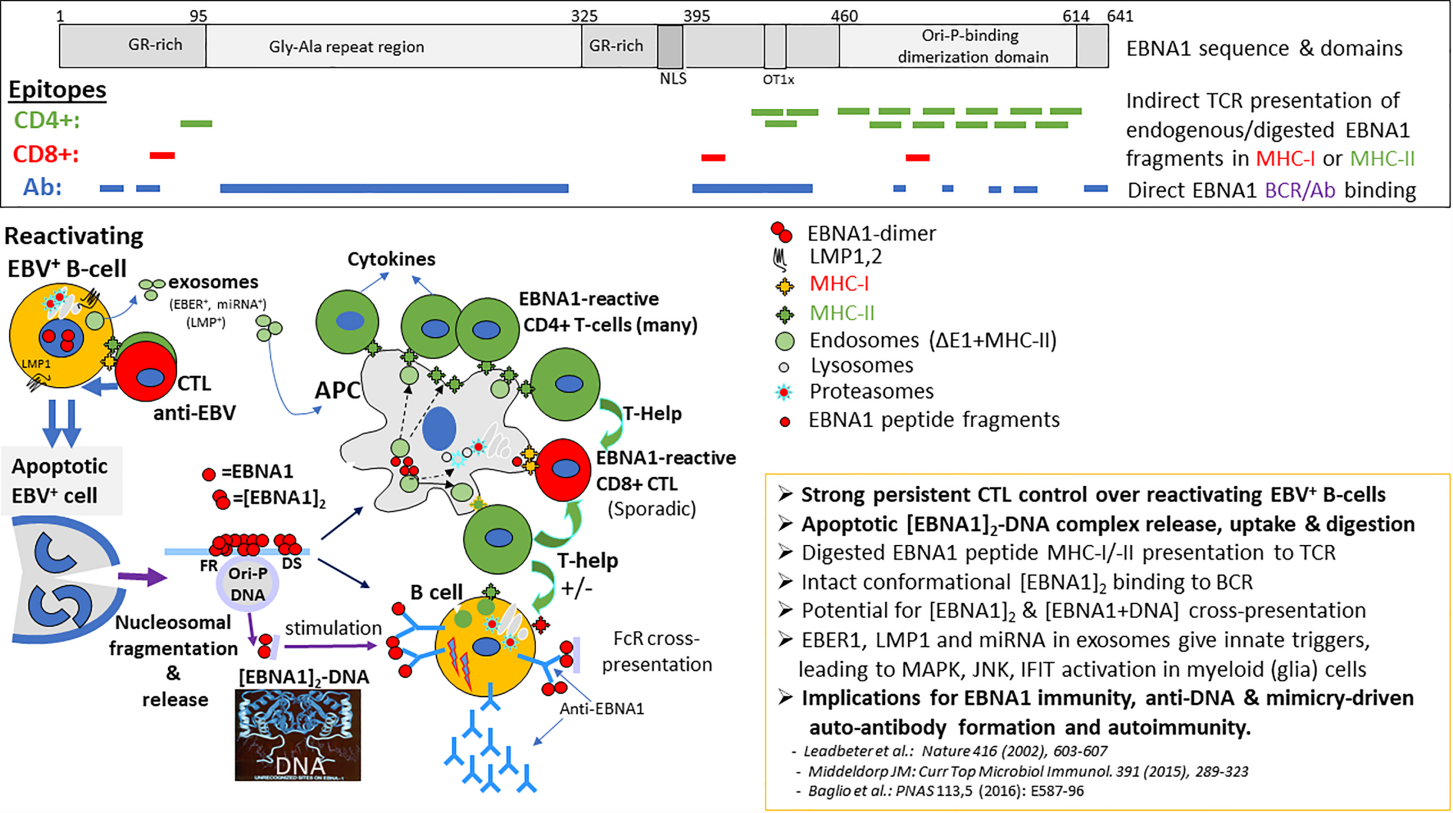
Processing, presentation and position of EBNA-1 epitopes for CD4+, CD8+ T-cells and antibodies (B-cells). Viable persistent and “reactivating” EBV infected B-cells secrete EBER1 and LMP1 containing exosomes in the head & neck lymphoid microenvironment, continuously activating resident myeloid cells (possibly including microglia and astrocytes) for innate inflammatory signaling and increased antigen-uptake, digestion and presentation. Such innate signals may attract and pre-activate T- and B-cells to the inflammatory site. In EBV carriers, lifelong strong adaptive CD4+ and CD8+ cytotoxic T-cell immunosurveillance exists against replicating latent (Latency-I,-II,-III) and reactivating (Latency-III, Lytic) EBV-infected B-cells recognizing viral peptides bound to surface MHC-I or MHC-II molecules, resulting in release of pro-inflammatory cytokines. This T-cell surveillance is causing repetitive apoptotic cell death of EBV-infected cells and release of nuclear content as apoptotic bodies, enhancing the local inflammatory milieu. Such apoptotic bodies contain EBNA1-DNA (and host protein) complexes which are taken-up by antigen-presenting cells (APC), digested and presented in MHC-II for further CD4+ T-help and cytokine activation. EBNA1-dimers and multimeric EBNA1-DNA complexes also bind to the B-cell receptor (BCR) on B-cells, directly triggering anti-EBNA1 antibody responses, supported by local CD40 T-cell interactions and cytokines (IL4, IL10, IL17A). Prevalent anti-EBNA1 antibodies may form (complement-containing) immune complexes with released EBNA1-dimer/-DNA resulting in Fc-receptor (FcR) uptake into APC’s and processing for MHC-II cross-presentation. Binding to B-cell receptor (IgM-IgD) directly triggers anti-EBNA1 antibody responses and possible anti-self autoimmune responses mediated by EBNA1-bound DNA and host nuclear proteins (i.c. ORC-complex) or *via* the mimicry domains within the EBNA1 sequence (see **Table 1**).

The serological evidence for EBV being a risk factor for MS is strong and growing, although the high levels of EBV seropositivity in adults make it hard to establish such association unequivocally. However, recent large-scale studies have revealed a near 100 percent EBV seropositive rate in MS patients, which is significantly higher compared to age-, gender- and population-matched controls (32, 33). The increased IgG seropositivity in MS patients particularly involves responses to proteins coded for by EBV latent genes (esp. EBNA-1) rather than lytic genes (EA, VCA), and is virus-specific because no elevated antibody levels to human cytomegalovirus (CMV) or Herpes simplex virus (HSV) are significantly linked to MS (32, 34). The risk of MS increased more than two-fold after a history of IM, as opposed to subclinical primary EBV infection (35, 36). The risk was further substantially elevated in individuals with an IM history and HLA Class II DR2b (DRB1*1501 β, DRA1*0101 α), which is the strongest genetic risk factor for MS (37). Furthermore, specific increases in serum anti-EBNA-1 antibody levels preceded the onset of clinically apparent MS by several years, showed increases during conversion from a clinically isolated syndrome (CIS) to definite MS and associated with active MRI lesions in established MS (38–41). Overall, anti-EBNA-1 antibodies are specifically elevated in MS and thought to originate from the periphery as the levels of anti-EBNA-1 antibodies relative to total IgG were higher in the serum compared to CSF in the majority of relapsing-remitting MS patients (34, 42). Importantly, EBNA-1 forms dimers and multimeric complexes tightly bound to viral and host DNA, which are released upon apoptotic death of EBV-infected host cells as induced by the lytic switch and/or anti-EBV cytotoxic T-cell (CTL) responses (see **Figure 2**). Such EBNA-1-DNA complexes are stable and highly immunogenic and human anti-EBNA-1 antibodies specifically recognize surface epitopes of such complexes (**Figure 2**, epitopes depicted in top section), but not the intramolecular dimer and DNA-interacting regions of EBNA-1 (43, 44). This suggests that anti-EBNA-1 antibody responses are driven by apoptotic EBNA-1-DNA complexes directly (via BCR on B-cells) or indirectly (via phagocytosis and/or Fc-/Complement receptor-mediated uptake and presentation in specialized antigen-presenting cells, APC) thus triggering anti-EBNA-1 T- and B-cell responses. Oligoclonal bands, which are found in the CSF of most MS patients, contain immunoglobulins that recognize EBNA-1 and intrathecal IgG from MS patients recognized defined EBNA-1 epitopes thus implying local anti-EBNA-1 antibody production that can lead to immune (complex)-mediated pathology (45). Interestingly, more recent detailed epitope mapping studies have confirmed the prevalence of anti-EBNA-1 IgG and IgM in CSF and sera from MS-patients, and revealed possible cross-reactive peptide mimicry epitopes, as will be detailed later (46, 47). The presence of high levels of anti-EBNA-1 antibodies and oligoclonal bands in CSF may reflect increased activation and locoregional multiplication with subsequent T-cell mediated apoptotic elimination of B-cells that are latently infected with EBV (26).

### Cellular Immune Responses Against EBV

EBV-reactive T lymphocytes are abundant in the circulation of EBV carriers and pivotal in controlling EBV homeostasis by eliminating undesired and potentially dangerous (re)activated EBV-infected B-cells (24). It is estimated that in healthy EBV carriers about 1% of all circulating T-cells are responsive to EBV-derived antigenic peptides, with latency-associated and immediate early lytic proteins being prime targets (23). CD4+ T cell responses are dominantly directed against EBNA-1, recognizing 12-15 mer peptide epitopes located within the stable EBNA-1/DNA dimer structure, suggesting complete complex degradation “*in trans”* in APCs (myeloid dendritic or B cells) before presentation on MHC-II to CD4+ T cells (48) (see **Figure 2**, epitopes in top section). On the other hand, CD8+ cytotoxic T cells (CTLs), which recognize MHC-I associated 8-mer peptides processed “*in cis”* by the host cell proteasome and presented on the surface of EBV infected cells, are much less abundant and very restricted in epitope recognition (24, 44, 49). Importantly, elevated EBNA-1 specific T-cell responses are detectable during initial stages (CIS) of MS and are predictive for symptomatic MS progression in parallel with anti-EBNA1 serology (40). Aberrant EBV-specific T-cell control has been found in MS patients, as will be detailed here below.

### EBV Persistence and Dysregulation of EBV Homeostasis in MS

Throughout life, EBV remains immunologically silent (*Latency-I*) in small numbers of B- cells (1 in 10^5^) in the blood, which home to the head and neck lymphoid tissues (15). EBV-infected B-cells periodically reactivate during lymph node passage to form new EBV+ B-cells (Latency-II/-III) or become plasma cells that may reproduce virus (lytic stage), leading to virus shedding in saliva and blood, but both types of reactivation are tightly controlled by CTLs to EBV latency-II/-III and immediate early lytic antigens to prevent B-cell lymphoproliferation (24). Importantly, physiological and immunological stressors can trigger inflammatory events that reactivate EBV from latency and drive multiplication of EBV-carrying B-cells (50, 51). Such aberrant state of EBV latency and/or reactivation proximal to the CNS may deregulate and enhance local (anti-EBV) inflammatory responses by release of exosomes (see **Figure 2**, lower section) containing EBV-encoded immunomodulatory RNAs (EBER1, miRNA) and proteins (LMP1). These exosomes may influence locoregional cellular functions and cytokine milieu (30, 31). CTL-mediated elimination of EBV+ B-cells may create apoptotic bodies containing EBNA1 that will be taken-up by local APC and induce further anti-EBV T-cell activation and associated (IL-17A) cytokine release. These locally produced cytokines are further affecting normal immune balances, triggering blood-brain-barrier (BBB) permeation and possible local oligodendrocyte (ODC) disfunction (52–54). Episodes of (natural or therapy-induced) immune suppression and T-cell dysfunction are clearly related to and provide evidence for the importance of a well-balanced immune control over EBV-reactivation and B-cell proliferation (17, 55). Several studies have addressed in detail the T-cell operators and viral targets involved in maintaining a life-long balanced immune control over EBV and their potential exhaustion and dysregulation in MS (24, 56, 57).

Aberrant T-cell responses in CSF and CNS have been widely associated with MS, and a dysregulated T-/B-cell interaction may be fundamental to MS pathogenesis (58–60). The trigger(s) for initiating and maintaining this dysregulated immune balance in the CNS remains elusive. Intrathecal EBV-specific T-cell responses against EBV and/or auto-antigens have been identified and may be involved in MS pathogenesis (61–65). These can reflect autoreactive EBV-infected B-cells entering the CSF from the blood (Pender’s hypothesis (66); and becoming activated locally, thus triggering inflammatory signaling and initiating EBV-specific or auto- (cross-) reactive CTL responses in the CNS. Alternatively, inflammation or stress-induced activation of (EBV-carrying) B-cells in meninges or locoregional lymphoid tissues may trigger cytokine responses and/or EBV encoded small-RNA (EBER)-induced inflammation *via* exosome secretion (**Figure 2**, lower section) that affect BBB integrity and allows anti-EBV immune cell passage and activation of microglia and astrocytes and dysfunctional of ODCs that together cause neuronal damage (2, 31, 51, 67–72).

Support for the hypothesis that anti-EBV inflammatory responses may be associated with the onset of MS came from reports of high levels of CTL activation against EBV but not CMV in the course of early MS (73). Studies on the characterization of CTLs to latent and lytic EBV antigens in relapsing-remitting MS (RRMS) found an expansion of CTLs specific for lytic EBV antigens (Zta/BZLF1 and BMLF1) during active disease in untreated MS patients but not in patients treated with natalizumab (74). Furthermore, the frequency of CTLs specific for EBV lytic and latent antigens was higher in active and inactive MS patients than in controls (74). More recently, activated EBV-specific CD8+ T-cells were found to predominate in CSF of MS patients compared to non-MS controls (63). Characterization of CD4+ T-cell responses in MS patients also showed dysregulation with strikingly elevated frequencies of EBNA-1-specific CD4+ memory T cells, with increased proliferative capacity and enhanced IFN-γ production (40, 75). T-helper (Th) cell responses to three other latent and three other lytic immunodominant EBV antigens and CMV epitopes did not differ between patients and controls suggesting that EBNA-1 specific Th1 cells in MS are capable of sustaining autoimmunity (75). Multiple auto-antigen targets have been defined for CNS-derived T-cells, some of which are specifically expressed on activated EBV-carrying B-cells and share MHC-II restricted antigenic epitopes with EBV proteins, in particular EBNA-1, as will be detailed later (63–65, 76).

### EBV Status and the MS Brain

Disruption of the BBB facilitates translocation of EBV-infected B-cells, inflammatory cytokines (i.c. IFN-1, TNF-α, IL17A), exosomes as well as anti-EBV antibodies and EBV-specific immune cells. One important question, which warrants further study, is how EBV and/or its products enter the MS brain during inflammatory events that (temporarily or chronically) permeate the BBB and whether such entrance is an early (triggering) or late (bystander) event in MS pathogenesis. As EBV resides in B-cells, which are able to traverse the “inflammation-damaged” BBB and traffic into CNS, its presence could be a mere bystander phenomenon. Recent studies indicate that epigenetic manipulation, for instance by inflammatory cytokines, can drive activation and CNS infiltration of EBV-infected B-cells (77). On the other hand, during periods of (EBV-related)? inflammation in locoregional lymphoid patches in the parenchyma, EBV+ B-cells and EBV-products in exosomes produced by such B-cells may cross the BBB and be taken-up by microglia cells to cause localized CNS inflammation (31, 78, 79). This would fit well with the characteristic MAPK^ERK^ activation status of microglia and astrocytes in MS lesions, which are not directly infected with EBV (2, 9).

The detection of EBV in the MS brain is not without controversy (80). Although initial PCR studies failed to find traces of EBV genome in MS-related CNS tissue biopsies (81), a 2007 report of abundant EBV infection and widespread EBV reactivation in acute MS but not in other inflammatory central nervous diseases triggered much attention (82). However, despite rigorous efforts, these findings could not be confirmed in subsequent studies by others (83–85). Additional studies demonstrated localized EBV-infection in both MS and control brains (86, 87). Early studies reported meningeal B-cells within specific structures, referred to as tertiary lymphoid follicles with a germinal center-like architecture and denoted as major sites of EBV persistence in the MS brain (82), which however were found mostly negative for EBV in other studies (83, 84). The reasons for these opposite findings may be due to technical issues, such as degradation of RNA, the use of different MS tissues, differences in reagents, and non-specific staining of EBER+ cells by *in-situ* hybridization leading to cytoplasmic instead of nuclear staining (80). More recent studies, using similar techniques have detected EBV in sporadic cells in 90% of MS cases compared to only 24% of non-MS cases with other neuropathologies with EBV-infection being present in microglia and astrocytes, besides B-cells (88). The presence of EBV-infected B cells in or near the CNS and the anti-EBV immune responses in CSF of MS patient implicates that EBV-related products released from “activated” infected cells (i.e. EBNA-1-DNA complexes and/or EBER-containing exosomes) may provide persistent triggers for local (neuro-)inflammation. Further detailed analysis of laser-cut CNS and meningeal tissue lesions suggested low-level EBV presence and prevalent activated EBV latency or restricted (abortive) viral reactivation capable of triggering localized anti-EBV T-cell responses in CNS of patients with active progressive MS (61, 87, 89).

If EBV is finally incriminated, how could latent infection play a role? Latent EBV-infection may activate innate immune responses and thereby drive neuroinflammation in the MS brain (86). The detection of EBER+ cells in active white matter MS lesions was linked to the overexpression of the innate cytokine interferon-α (IFN-α) by cells with the morphology of microglia and macrophages (86). IFN-α is part of the type-I interferon family and an important player in anti-viral immunity. Its biological effect is distinct from IFN-β, which is also used to treat MS as discussed below. Latent EBV infection may contribute to neuroinflammation by triggering IFN-α production, as supported by the findings that EBERs can bind to Toll-like receptor 3 and potentially other intracellular receptors such as retinoic acid-inducible gene 1 (RIG-I) and elicit IFN-α production (31). However, these findings were not exclusive to the MS brain, as EBER+ cells were also found in cases of stroke and CNS lymphoma (86). This is also supported by more recent studies (87). Interestingly, recent studies have revealed a link between innate iNKT cells and B-cell responses, which would substantiate the potential role of EBV-derived inflammatory products (EBER-1 in exosomes, EBNA-1-DNA complexes) as triggers for dysregulated (autoimmune) B-cell responses (90). Whether MS-triggering EBV activity is located within the CNS or its boundaries remains to be determined, as well as whether such EBV activity is an MS initiating event or a consequence of (autoimmune)? inflammation.

### EBV Status and Lymph Nodes in MS

Despite the dogma that the CNS has “immune privilege” to mitigate immunological damage to neurons, neuroimmune interactions may play an important role in the MS brain. There is increasing evidence for an intimate interaction between the brain and the immune system in the dural sinuses (91). Lymphatic vessels in the meninges provide an important link between the CNS and peripheral immune system and can play a role in autoimmunity in MS (91). Vessels in the dura mater that drain CSF from the brain to the cervical lymph nodes and pathogens in lymph nodes can lead to the initiation of immune responses. B-cells seem to traffic freely across the tissue barrier, with the majority of B-cell maturation occurring outside of the CNS in the secondary lymphoid tissue (92). Furthermore, CNS B-cells have access to lymphoid tissue where they may encounter antigen and experience activation and affinity maturation.

There is evidence to suggest that cervical lymph nodes may serve as a latent viral reservoir due to dysregulated EBV activation (61, 87, 89). Histopathological findings of MS tissue from cerebral hemisphere, brain stem, and cervical lymph node of a patient with primary progressive MS (PPMS), who died of an ischemic stroke, showed EBER+ cells within B-cell follicles in the paracortex of the cervical lymph node (93).

### EBV and Molecular Mimicry in MS

Autoimmunity can be caused by TCR-independent bystander mechanism or by cross-recognition of autoantigens when antigenic epitopes shared between a pathogen and host generate a cross-reactive B- or T-cell response that breaks self-tolerance and causes antibodies or T-cells respectively to damage host tissues. The concept of molecular mimicry at the level of T-cells causing the autoimmune disease was first elaborated by Ebringer in 1979 (94). A molecular mimicry hypothesis for MS has been proposed, whereby epitopes in viral pathogens, such as EBV, endogenous retroviruses, HHV-6 cross-react with epitopes in brain proteins and elicit cross-reactive B- or T-cell responses, which create inflammatory triggers and immunopathology (95–97).

EBV is thought to lead to marked immune activation and stimulate autoreactive T-cells *via* molecular mimicry between foreign agents and myelin peptides (98). These findings are supported by mechanistic studies into the structure of the T cell receptor (TCR) from an MS patient, which recognized both a DRB1*1501-restricted myelin basic protein (MBP) peptide and a DRB5*0101-restricted EBV peptide. Both HLA-peptide complexes revealed a marked degree of structural similarity at the surface presented for TCR recognition, which provided structural evidence for molecular mimicry involving Class-II HLA molecules (99). Recent studies in a humanized mouse model confirm a central role for HLA-DR15 restriction elements in causing aberrant anti-EBV immune responses with risk of autoimmunity (100).

Prior studies addressing the possibility of molecular mimicry in MS found clonally expanded EBNA-1-specific CD4+ T-cells that potentially contributed to the development of MS by cross-recognition with myelin antigens (75, 101). MS patients showed increased T-cell responses to EBNA-1 but not to other EBV-encoded proteins nor to other viruses such as influenza, HSV-1 and CMV. An expanded reservoir of EBNA-1-specific central memory CD4+Th1 precursors and Th1 (but not Th17) polarized effector cells recognized myelin antigens more frequently than other autoantigens that are not associated with MS (101). More recent findings shed light on how EBV may trigger mimicry-based autoimmunity, suggesting a crucial role for EBNA-1 in inducing mimicry-based self-reactivity as summarized in **Table 1** and visualized in **Figure 2**. Antigenic products induced by EBV within (e.g. alpha B-crystallin (CRYab) (105, 106, 114), or released from EBV infected B-cells (i.c. EBNA-1-DNA, LMP1,2 in exosomes, Lytic phase components) are thought to activate HLA-DR/peptide reactive CD4+ T-cells, which then respond to potentially “pathogenic” self-peptides or autoantigens with EBV-related amino acid sequence homology (= mimicry) such as myelin basic protein (MBP), myelin oligodendrocyte glycoprotein (MOG), neurofilament light chain (NFL). Multiple putative cross-reactive epitopes in human and EBV proteins interacting with IgG from CSF or serum from MS patients have been identified (**Table 1**). A recently identified pathogenic self-peptide is derived from the RAS Guanyl Releasing Protein 2 (RASGRP2), which is expressed by B-cells but also in neurons and other brain cells. B-cells presenting RASGRP2-derived peptides are thought to trigger autoreactive T-cells, similar to CRYab (65, 76, 102, 104, 114). These HLA-DR-self peptide-reactive CD4+ T-cells can cross the BBB and enter the brain where they orchestrate an autoimmune attack by producing inflammatory mediators leading to demyelination and axonal injury.

**Table 1:** Overview of viral proteins involved in antigen mimicry in MS.

Virus	Viral protein	Self-protein	Nature of cross-reaction	Study	Reference
**EBV**	EBNA-1	β-Synuclein	HLA DR2b binding (potentially CD4+ T-cells)	Ramasamy et al. (2020),	(102)
	EBNA-1	α-Synuclein, CRYAb, MBP, MOG & neurofilament light chain	Cross-reactivity of anti-EBNA1 peptide-specific antibodies with human brain protein extracts and purified brain proteins and identification of sequence homologies	Vertelman & Middeldorp (unpublished), Middeldorp (2015)	(44)
	EBNA-1	MBP	Serum antibodies in MS patients	Jog et al. (2020)	(103)
	EBNA-1	Mix of myelin proteins	CD4+ T cells	Lünemann et al. (2008)	(101)
	EBNA-1	Anoctamin 2	Antibodies in MS patients	Tengvall et al. (2019)	(104)
	EBNA-1	Alpha-B Crystallin (CRYAb)	Serum and CSF antibodies in MS cases EBV+ LCL and MS-CNS lesions	Hecker et al. (2016) Van Sechel et al. (1999) Van Noort et al. (2010)	(46) (105) (106)
	EBNA-1	hnRNP-L	Serum antibodies in MS	Lindsay et al. (2016)	(107)
	DNA-polymerase	MBP	CD4+ T-cells	Wucherpfennig & Strominger (1995)	(98)
	BFRF3 (VCA-p18)	Septin-9	Antibodies in MS patients	Lindsey (2017)	(108)
	BRRF2	Mitochondrial antigens	Serum antibodies in MS	Dooley et al. (2016)	(109)
	LMP-1	MBP	CSF antibodies in MS and Mouse immunizations	Lomakin et al. (2017)	(110)
	LMP-1	a-Synuclein	Monoclonal antibody on human brain tissue	Woulfe et al. (2016) Middeldorp, unpublished	(111)
	Multiple	Multiple	Pentapeptide epitope homology	Kanduc and Shoenfeld, (2020)	(112)
	BZLF1	Unknown	CD8+ T-cells in MS brain	Serafini et al. (2019)	(61)
**HHV-6**	U24	MBP	CD4+ T-cells and antibodies in patients	Tejada-Simon et al. (2003)	(113)
**pHERV-W**	env	MOG	HLA DR2b binding (potentially CD4+ T-cells)	Ramasamy et al. (2020)	(102)

A structural approach to investigate molecular mimicry identified a structurally related pair of peptides from EBNA-1 and β-synuclein, a brain protein implicated in MS (102). Binding experiments showed the binding of the predicted peptides to HLA DR2b with characteristics comparable a with the well-known Th-epitope in myelin basic protein (MBP). Structural modeling of the peptides with HLA DR2b revealed binding to the peptide-binding cleft similar to MBP and relative conservation of the surface exposed, potential TCR contact residues in the two peptides. This suggests that molecular mimicry is possible between the EBNA-1 and β-synuclein peptides.

A recent study presented a case for molecular mimicry between Anoctamin 2 (ANO2) and EBNA- 1 associates with MS risk (104). ANO2 is a chloride channel protein expressed in the CNS. MS patients showed increased autoantibody reactivity to ANO2 with high sequence similarity between epitopes in ANO2 and EBNA-1 (**Table 1**).

Whether antigenic mimicry is a cause or consequence of MS-related inflammation and is driven by the mimicry-domains in EBNA-1 remains to be established.

### EBV and Reactivation of Human Endogenous Viruses

The regular (stress-triggered) reactivation of EBV in or near the CNS with associated activation of host genes and release of EBV-encoded components, together with the loco-regional (anti-virus/-self) inflammatory cytokine responses triggered by and against EBV, may set-of a number of cellular signaling events in actively responsive as well as bystander cells (B-/T-cells, glia-astrocytes, ODCs). These EBV-induced molecular events may lead to changes in transcriptional control/activation of viral sequences encoded within the germline genome, in particular endogenous retroviruses (HERV) and integrated HHV6A (each individually detailed further below). Well known examples are the induction of HERV-K18 (115, 116) and HERV-W (117) gene-products by EBV infection in B-cells and/or cytokine-driven reactivation (118) of these endogenous viruses. The induced HERV-K18/-W proteins may serve as “superantigens” further inducing polyclonal Vβ TCR-driven activation and associated cytokine releases (119). On the other hand, it has been described that endogenous retroviral elements (i.c. HERV-W-env (Syncytin-1) may enhance lytic viral gene expression of gamma-herpesviruses in latently infected B-cells, making the interaction two-sided (120). Dysregulated (virus- or cytokine-induced) expression of HERV-K/-W elements have clear implications for the pathogenesis of multiple neurological disease, including MS (121), secondary to the initial triggering by EBV-driven mechanisms. Of relevance is the possible induction of mitogen-activated protein kinase extracellular signal-regulated protein kinase (MAPK-ERK) (122) pathway in HERV-W-ENV expressing cells, which is characteristic of MS glia-cell activation (9). Together, these observations strengthen the 3-viral cascade hypothesis (123), with EBV as leading pathogen (**Figure 1**) and comprising a multi-factorial interaction of virus-, altered self or mimicry- and cytokine-driven inflammatory pathogenic events underlying the different manifestation of MS disease, being non-progressive (CIS), reiterating (RRMS) and progressive (SPMS, PPMS) with increasing neurological damage.

### Future Studies on the Relationship Between EBV and MS

Defining the roles of autoimmune responses as cause or consequence in the pathogenesis of MS and their potential link to viral antigen mimicry and auto-reactive EBV-infected B-cells merits further analysis. This also holds for the putative inflammatory role(s) of EBV-derived components (EBERs in exosomes and/or EBNA1-DNA complexes) in triggering loco-regional activation of the MS-characteristic inflammatory MAPK pathway in microglia (2, 9). The role and mechanism of EBV and cytokines on activating other endogenous viruses, such as HERVs and HHV-6 also deserves attention (9). Since all MS patients are EBNA-1-IgG positive, the involvement of EBNA-1-IgG immune complex formation and inflammation near CNS should be further analysed. Single cell sorting could be utilized to provide more definitive proof on the presence and status of EBV (analysis of viral DNA and RNAs) in the MS brain and nearby regional lymphoid tissues (61, 89). Frozen tissue containing (pre-)active HLA-DR+ lesions with reactive glia-cell and ODC-clusters from MS cases will be needed to perform single-cell B cell receptor (BCR) and TCR sequencing as was recently done on Alzheimer’s patient biopsy material (124). Cell types can be selected based on cell-specific protein markers and many features may be used simultaneously to classify cells by molecular analysis (125). EBV-specific TCRs have been detected in the CSF from patients with Alzheimer’s disease. However, these data are not direct evidence of a causal link between EBV and Alzheimer’s disease (124). A search for EBV-DNA, EBER1-RNA, EBV encoded miRNA or EBNA-1/LMP-1 in combination with single cell analysis of CNS cell type-specific RNA, T-cell lineage and TCR sequence has not yet been conducted in in MS patients to our knowledge, but methods to do so are being developed (126, 127). Similarly, the presence, frequency and level of HERV-K/-W RNA as well as HHV6A gene-expression in defined cell types in MS-related (early) CNS lesions requires further analysis. Other interesting topics include the link between bacterial infection (periodontitis, inflammatory bowel disease, etc.), short-chain fatty acids, and vitamin D status or “stress” factors and their influence on (chronic or repeated) EBV reactivation in causing neuroinflammation, exemplified by the well-defined effects of butyric acid, butyrate derivatives and glucocorticoids on ODC activation, triggering B-cell proliferation and switching EBV from tight latency into reactivation and lytic replication (128–131).

### Therapeutic Approaches Targeting EBV-Infection in MS

Efforts to develop antiviral strategies for treating MS are underway. Interferon-β (IFN-β) is one of the first-line treatments of MS and an important player in antiviral immunity. However the mechanism by which the therapeutic effect takes place remains somewhat elusive to date. It is thought that IFN-β has antiproliferative effects and down-regulates T-cell activation by altering the expression of proteins involved in antigen presentation, and promotes the differentiation of activated T cells away from a Th1 response (pro-inflammatory) and towards a Th2 response (anti-inflammatory). Clinically effective IFN-β therapy was associated with a downregulation of proliferative T-cell responses to EBNA-1 and showed efficacy in reducing pHERV-W and HHV6-A plasma viral loads, two additional viral risk factors in the context of MS, as will be described further below (132, 133).

New monoclonal antibody-based treatments targeting B-cells have recently been introduced and proven highly successful in reducing MS clinical symptoms. The idea that these treatments impact on the B-cell-tropic EBV warrants further study (8, 134). The B-cell depleting antibody ocrelizumab significantly reduced annual relapse rate and dramatically limited the appearance of new Gadolinium-enhanced T2 lesions, as well as disability progression (135). However, this treatment proved less effective in more advanced progressive stages of MS and is not considered curative. The use of haemopoietic stem cell transplantation (HSCT) has been suggested as curative MS-treatment aiming at the elimination of pathogenic reactive lymphoid cells and to re-boost the immune system. Interestingly, autologous HSCT may deplete EBV from the pathogenic equation, as early studies indicated complete elimination of endogenous EBV by HSCT (136). However, EBV-elimination is not guaranteed after HSCT and the HLA-DR based genetic susceptibility for MS remains, whereas the health risks associated with HSCT are considerable and HSCT may not be suitable for all categories of MS (137).

Studies examining the effect of antiviral and antiretroviral drugs on MS disease activity are currently planned. In past antiviral trials targeting herpes virus, treatment only reduced lytic viral replication without affecting latent virus. Several nucleoside analogs have been shown *in-vitro* to impact EBV lytic replication including acyclovir and penciclovir, ganciclovir and tenofovir (138). A case report described the resolution of MS symptoms, which remained subsided for more than 12 years (139), in a MS patient diagnosed with HIV after starting HIV antiretroviral therapy. It is interesting to speculate to what extent the observed benefits reflect an impact of anti-retroviral therapy on neuroinflammation and on EBV-mediated disease mechanism.

A novel strategy is currently tested to eliminate EBV-infected B-cells using an EBV-specific CD8+ T-cell therapy. Autologous or allogeneic infusion-cell therapy studies by Pender and colleagues have focused on inducing CTL activity against latent EBV proteins (140). A phase-1 trial of autologous EBV-specific T-cell therapy in progressive MS showed short-term clinical improvement in 7 out of 10 patients and noted no serious adverse effects. The patients were treated with four escalating doses of *in vitro*-expanded autologous EBV-specific T-cells targeting EBNA-1, LMP1 and LMP2A. Clinical improvement following treatment was associated with the potency of EBV-specific reactivity of the administered T-cells. However, the beneficial effect was sustained in a limited number of cases in this trial (141).

Targeting neuroinflammation *via* Fc- (FcR), B-cell (BCR) and Toll-like receptor signaling may also be achieved with Bruton’s tyrosine kinase (BTK) inhibitors. BTK is a signaling molecule involved in maturation and activation of B-cells through BCR and FcR. BTK has been demonstrated to also play an important role in signaling pathways of multiple Toll-like receptors (142, 143). The BTK inhibitor AG126 has recently been tested in experimental autoimmune encephalomyelitis (EAE), the animal model of MS, and reduced clinical symptoms, immune cell infiltration in the CNS, microglia activation and myelin damage, and decreased Th17 differentiation. BTK inhibitors also impacted LMP2A mediated IL-10 production crucial for EBV survival by increasing STAT3 phosphorylation *via* a PI3K/BTK-dependent pathway (144). A recent clinical trial examined the BTK inhibitor evobrutinib in a phase 2 clinical trial in MS. Patients with RRMS who received 75 mg of evobrutinib once daily had significantly fewer enhancing lesions during weeks 12 through 24 than those who received placebo (145). However, there was no significant difference with placebo for the other dosing regimes, nor in the annualized relapse rate or disability progression at any dose. A second oral BTK-inhibitor tolebrutinib, capable of passing the BBB, was also evaluated in a phase-2b trial with similar beneficial effects for the highest 60 mg dose with minimal side-effects (146). Newer BTK inhibitors are being developed, which more specifically inhibit BCR and FcR mediated signaling in B-cells and myeloid cells, which may have impact on the proposed reactive T-/B-cell and EBV-EBNA1 antigen-driven inflammation in MS lesions (147). Alternative options to reduce neuroinflammation in MS include inhibition of fibroblast growth factor receptor and MAPK-signaling (9, 148, 149) or use of ursolic acid, a well-tolerated oral drug that reduced neuroinflammation and stimulates remyelinisation (150).

Suppressing the function of EBNA-1, as crucial viral gene for EBV DNA maintenance in B-cells, is being tested for treatment of EBV-driven cancers, including lymphomas and carcinomas. Such an approach may also prove relevant for MS treatment, provided that EBV within B-cells indeed is a master player in MS pathogenesis (151, 152).

## Human Herpesvirus 6 and MS

### A Brief Description of HHV-6

Human herpesvirus 6 (HHV-6) is a ubiquitous β-herpesvirus associated with a number of clinical disorders including MS. Two closely but biologically distinct variants (HHV-6A and HHV-6B) have been described with different tropisms. Although some authors have described a possible relation between HHV-6B and MS (153), HHV-6A is more strongly associated with MS (154, 155). HHV-6 has a seroprevalence rate of 70 to 90 percent in the human population (156). Most adults become infected as infants and have seroconverted by the age of two. There have been reports of neuroinvasion and persistence of HHV-6 in children with neurological complications, e.g. febrile seizures and encephalitis (156, 157). The virus can reactivate, especially in cases of immune deficiency, such as in acquired immune deficiency syndrome (158). HHV-6 also has the capacity to be inherited in a Mendelian fashion in up to one percent of the population as chromosomally integrated HHV-6 in which the complete HHV-6 genome is integrated into the telomere of every chromosome (159).

### HHV-6 Infection and MS

A question of interest is how HHV-6 can cause or contribute to neuroinflammation in MS as it is considered a risk factor for MS (160). New animal models have recently been established, mainly for HHV-6A and reproduced some pathological features seen in humans. Animal models have been slow to develop because rodents lack CD46, the receptor for cellular entry of the virus. It is hypothesized that HHV-6 can modulate the functions of the CD46 receptor by binding to it and levels of soluble CD46 are increased in the serum of patients with MS (161). Studies in a CD46 transgenic murine model of HHV-6A infection described persistent infection of the brain (162) with infiltrating lymphocytes in periventricular areas of the brain indicative of neuroinflammation. HHV-6A triggered chemokine and cytokine production *via* stimulation of Toll-like receptor 9. The marmoset model showed that animals inoculated intravenously with HHV-6A exhibited neurologic symptoms, while marmosets inoculated with HHV-6A intranasally stayed asymptomatic (163). Other studies showed that HHV-6A can enter the CNS *via* the olfactory pathway (164).

Several groups have reported the presence of HHV-6A in MS plaques and normal-appearing white matter within the MS brain as well as in normal controls (155, 165). A groundbreaking report in 1995 obtained evidence that HHV-6 was a common commensal virus of the brain expressed in neurons and glial cells (165). Expression of HHV-6 antigens was observed in oligodendrocytes in MS cases, but not in various controls. Moreover, in MS patients, nuclear HHV-6 staining in oligodendrocytes was most commonly associated with MS plaques. These findings were later confirmed and HHV-6A genome-containing cells, including ODCs, were detected in biopsy specimens of acute MS lesions (166). In addition, an association was found between HHV-6A reactivation and disease activity in RRMS and secondary progressive MS (SPMS) (167). The increase of the anti-HHV-6A/B IgG and IgM titers predicted clinical relapses and highlighted their usefulness as disease biomarker of clinical response to the different disease-modifying treatments (DMTs) (155). In addition, increased IgM serum antibody responses to HHV-6 early antigen (p41/38) were detected in patients with RRMS when compared to patients with primary progressive MS (PPMS), SPMS, patients with other neurologic disease, patients with other autoimmune diseases, and normal controls (168).

### HHV-6 and Molecular Mimicry in MS

Molecular mimicry between HHV-6 and brain proteins has been suggested and may help understand the potential role of HHV-6 infection in the activation of autoimmunity and its implication in the pathogenesis of MS (**Table 1**). Sequence similarity between MBP residues 93-105 and the U24 protein of HHV-6 has been identified (113). The precursor frequency of cross-reactive CD4+ T-cells recognizing peptides from MBP and U24 were significantly elevated in MS patients compared to healthy controls.

HHV6A has also been found to activate a HERV-K18-encoded superantigen, which in turn activated T-cells carrying receptors of the Vβ13 family (169). T-cell clones, activated in this way, had TCRs that recognized the immunodominant encephalitogenic MBP peptide (residues 83-99) presented by HLA DR2b, thereby demonstrating the potential for causing immunopathology in HLA DR2b-positive MS patients (113).

### Transactivation of EBV and HERV by HHV-6

Transactivation can be triggered by viral proteins, also called transactivators, which act in trans (i.e. intermolecularly) in the same co-infected host cell. The transactivation of EBV by HHV-6 was described whereby HHV-6 upregulated the immediate-early EBV Zebra gene transcription through a cyclic AMP-responsive element associated with the Zebra gene (170, 171). Additional studies showed that HHV-6 variant A, but not variant B, infected EBV+ve B-cells activated the endogenous latent EBV genome through a BZLF-1-dependent mechanism (172).

HHV-6A also transactivated other viruses. HHV-6A and HHV-6B induced transcriptional activation of the endogenous retroviral superantigen HERV-K18 (169, 173). An interesting recent study reported that HHV-6A increased the expression the envelope protein of a pathogenic version of HERV-W in human glial cell lines, supporting the hypothesis that HHV-6 infection may promote neuroinflammation (174).

## Human Endogenous Retrovirus W

### A Brief Description on HERVs

Human endogenous retroviruses (HERVs) are remnants of ancient RNA viruses that have DNA copies of their genome incorporated into the human genome (175). HERVs compose approximately 8% of human DNA, although many HERVs have undergone loss of function mutations in critical genes or become highly truncated (176, 177). The possible roles of different HERV family members in MS have been comprehensively reviewed recently, including how immune activation, inflammation, and oxidative stress can influence the transcription of HERV genes (178). HERV-W family members have particularly attracted attention. Among the 13 HERV-W loci with full-length *env* genes coding for viral envelope proteins in the human genome (179), only a single gene located in chromosome 7q21.2 and coding for Syncytin-1 has an uninterrupted open reading frame. Syncytin-1 has evolved or undergone exaptation to perform an important fusogenic function in human placentation in the fusion of cytotrophoblasts to form the placental syncytiotrophoblast (180). Another HERV env protein termed Syncytin-2 from a different HERV family, HERV- FRD, has also been similarly exapted to play an essential fusogenic role in forming the placental syncytiotrophoblast (181).

### HERV-W Envelope Proteins and MS

An MS-associated retrovirus termed MSRV, also a member of the HERV-W family, has been particularly implicated in MS because virus particles and reverse transcriptase activity were detected in MS patients (182). MSRV env protein has been found to be present in microglia associated with myelinated axons in MS lesions, and implicated in inflammatory myelin and neuron damaging activity by microglia *in vitro* (183). A humanized IgG4 monoclonal antibody to MSRV env has been reported in a clinical trial to show a neuroprotective effect in RRMS (184). MSRV env has also been detected in macrophages, astrocytes and infiltrating lymphocytes within lesions (185). The MSRV env is 87% identical to Syncytin-1 in amino acid sequence by BLAST analysis (95), and to clearly differentiate the two proteins and their origins, which has in the past caused confusion in the literature, MSRV is now referred to as pathogenic HERV-W or pHERV-W (183). The genomic origin of the pHERV-W env protein remains a puzzle in view of the absence of a full-length gene for a HERV-W env protein other than Syncytin-1. However, it has been proposed that pHERV-W env may be derived from a HERV-W gene on the X chromosome at Xq22.3, which has a premature stop codon at position 39, through a process of somatic mutation or trans-splicing (186).

HLA Class II DR2b (composed of the DRB1*1501 β chain that pairs with a relatively invariant DRA1*0101 α chain) is the strongest genetic risk factor for multiple sclerosis (187). pHERV-W env, Syncytin-1 and Syncytin-2 on one hand and the three myelin proteins that are principal targets of an autoimmune response in multiple sclerosis (MBP, PLP and MOG) showed sequence similarities between potential Th cell epitopes within pairs of viral and myelin peptides predicted to bind HLA DR2b (95). A set of the sequence homologous peptides from pHERV-W env, Syncytin-1, Syncytin-2, and MOG were shown to bind to HLA DR2b molecules in an *in-vitro* assay (102). These results were consistent with a molecular mimicry hypothesis (95, 102) that brings together the genetic (i.e. HLA) and viral (i.e. HERV-W) factors that influence susceptibility to MS (**Table 1**). Furthermore, it is speculated that EBV may provide the necessary immune activation and inflammatory stimuli that permits transcription of the pHERV-W env gene (95, 102), and also that the EBV protein EBNA-1 may provide the trans-splicing activity needed to synthesize the full-length pHERV-W env protein (102). It has also been postulated that HHV-6 may synergize with pHERV-W to initiate MS (174).

### Syncytin-1, Syncytin-2, and MS

Syncytin-1 binds to the Na-dependent amino acid transporter-1 and -2 (ASCT1 and ASCT2) (188), which are also expressed in neurons and glia (189). It has been suggested that Syncytin-1 expression increases in monocytes during infections and MS relapses, two conditions reflecting inflammation (119). Syncytin-1 has been reported to be up-regulated in activated lymphocytes, monocytes, and effector NK cells, suggesting a role in the first steps of immune cell activation (119). However, these studies used a commercial antibody against an unknown peptide of unspecified length from the N-terminus of the Syncytin-1 protein, and it is possible that this antibody recognizes pHERV-W env instead of Syncytin-1 in the targeted tissues because of the 87% amino acid sequence identity between the two proteins. Therefore, further clarification is needed on whether these studies (119) detect and differentiate pHERV-W env and Syncytin-1.

However, the immunological role of potential MOG-cross-reactive Th cell epitopes potentially present in Syncytins-1 and -2 are an enigma that needs resolution. It is possible that these epitopes in these two ‘natural’, and therefore potentially tolerogenic, human proteins may have an immune regulatory role in preventing molecular mimicry-led immune damage. The 87% amino acid sequence identity between Syncytin-1 to pHERV-W env and the 38% amino acid sequence identity between Syncytin-2 and pHERV-W env by BLAST analysis make further study of Syncytin-1 and -2 important. Both Syncytins-1 and -2 functions have immunosuppressive functions (190). Syncytin 2 decreased Th1 cytokine production (191), and Syncytin-1 inhibited production of TNF-α, IFN-γ, and CXCL10 (192). However, such immunosuppressive functions have not been demonstrated for pHERV-W env, which instead shows inflammatory properties (183, 185, 192). The basis for the differences in immunological properties of three homologous proteins pHERV-W env, Syncytin-1 and Syncytin-2, however, remain to be fully elucidated. Differences in fusogenic functions are related to differences in amino acid sequences because only Syncytin-1 and Syncytin-2, and not pHERV-W env, have sites for cleavage by the protease furin that is necessary for initiating membrane fusion of cytotrophoblasts to form syncytiotrophoblasts in the placenta (190). The putative 16-amino acid immunosuppressive domain of Syncytin-1 differs from the corresponding sequence in pHERV-W env by a charge-altering glutamic acid to lysine change (102) that may eliminate immunosuppression. Differential binding of monoclonal antibodies to Syncytin-1 and pHERV-W env demonstrate antigenic differences and there also differences in membrane localization and oligomerization properties of the two proteins (193). Furthermore, Syncytins 1&2 are mainly expressed in the developing placenta and are also present as components of placental exosomes formed in a tolerogenic environment, while pHERV-W env is known to be produced in an inflammatory environment in the CNS (183, 185). These differences between the two Syncytins and pHERV-W env may be pertinent to their varying roles in the etiology of MS, and require further investigation.

### pHERV-W and MS

As virus particles displaying reverse transcriptase activity, pHERV-W has been particularly implicated in MS (194–196). It has been shown that the expression of the pHERV-W env gene product is significantly elevated in brain lesions in MS plaques and associated with the extent of active demyelination and inflammation (171, 172, 174, 175). pHERV-W can induce T-cell responses and pro-inflammatory cytokines release (197, 198). Sequencing pHERV-W in MS prompted the initial link between the HERV-W family and MS (199). pHERV-W mediates T-cells to cause neuropathology *in-vivo* (200). The HERV-W gene at Xq22.3 has been suggested as the potential cause for the higher prevalence of MS in women. However, the reported role of pHERV-W load in peripheral blood mononuclear cells (PBMCs) as a biomarker for MS needs more investigation (201).

During efficacious therapy with IFN-β, a longitudinal evaluation of patients revealed that viremia fell rapidly below detection limits; notably however, one patient, after initial clinical and virological benefit, had pHERV-W rescue, preceding strong disease progression and therapy failure (202). It was suggested that the evaluation of plasma pHERV-W could be considered the first prognostic marker for the individual patient to monitor disease progression and therapy outcome (203). A study of patients with optic neuritis, a disease frequently prodromic to MS, makes this possibility stronger as patients had significantly higher pHERV-W positivity than control groups (203).

### Approaches Targeting pHERVs MS

One approach focusing on the postulated role of pHERV-W in the etiology of MS has been to initiate clinical trials with temelimab, an IgG4 humanized monoclonal antibody against the proinflammatory pHERV-W env (184, 204). Additional first attempts have been made in a clinical study with the HIV integrase strand inhibitor, raltegravir, which did not impact on disease activity but found interesting correlations between HERV-W markers, EBV shedding and new MRI lesions, independent from treatment effects (205). Other attempts are being made to induce tolerance and/or induce regulatory T- cells in MS, against specific encephalogenic peptide epitopes. Tolerogenic dendritic cells pulsed with peptides shown promise in preliminary clinical trials (206). Novel approaches have shown promise in mouse models of EAE for inducing regulatory CD8+ T cells (207) and regulatory CD4+T cells (208).

## The inflammatory Cascade in MS Pathogenesis

Our understanding of the underlying immunopathology of MS is still incomplete. We propose that EBV, pHERVs and HHV-6A are part of an inflammatory cascade with mimicry-driven autoimmunity contributing to the pathogenesis of MS in genetically susceptible individuals (**Figure 1**). Based on the strong epidemiological link between EBV and MS our hypothesis predicts a leading pathogenic role for EBV and its products (**Figure 2**) in triggering CNS-localized inflammatory lesions characteristic of MS. This is paralleled by endogenous virus reactivation and interaction between the 3 viruses within and beyond the CNS-proximal immune system and points to testable pathogenic parameters and targeted treatment options.

In summary: EBV-infected B-cells in Latency-I programme circulate in blood and home to head and neck lymphoid tissues near the CNS, especially to meninges or brain lymphatics linked to Ring of Waldeyer lymphoid system, including tonsils (209–211). The EBV genome gets activated during passage through these lymphoid structures to replicate and produce new Lat-II/III B-cells (proliferative blast stage) that switch to the resting stage again (Lat-I/-0) when leaving lymphoid structures and re-entering the circulation (25). However, defined epigenetic triggers including hormonal stress factors, other infections (bacterial or viral) and related products or induced inflammatory cytokines may lead to EBV lytic reactivation and/or uncontrolled proliferation of EBV-infected B-cells and release of EBV products like EBNA-1-DNA complexes and EBER-exosomes, thus inducing or enhancing local inflammation and antigenic cross-presentation (**Figure 2**). EBV-infected B-cells are normally successfully eliminated by EBV-specific T-cells, however, overstimulation and uncontrolled proliferation may induce a state of T-cell exhaustion as seen in infectious mononucleosis, X-linked lymphoproliferative syndrome and HIV infection, allowing EBV-positive B-cells to escape T-cell surveillance (8, 56, 57, 62). Overactive EBV-Lat-III B-cells may then trigger further local inflammation and activation of endogenous pHERV-W/-K and HHV-6A in regional virus- or cytokine-activated cell types (lymphocytes, microglia, astrocytes and oligodendrocytes (**Figure 1**). This localized inflammation impacts on the integrity of the blood-brain barrier and facilitates translocation of (EBV-infected) B-cells, inflammatory cytokines, exosomes as well as anti-EBV antibodies (esp. anti-EBNA1), immune complexes and EBV-specific immune cells.

This basically EBV-driven process may lead to the activation of CNS-resident myeloid cells (microglia, astrocytes) into an M1-state (2, 9) and triggering pHERVs and HHV-6 together driving auto-reactive immune responses and damage to CNS-resident microglia and ODCs leading to neuronal damage by targeting and other neural self-antigens *via* molecular mimicry (**Table 1**). By eliminating EBV from the equation (i.c. by HSCT, anti-B-cell or anti-EBV T-cell therapy), and by inhibiting specific receptor-driven signaling (BTK, MAPK-ERK, JAK/STAT) or inducing the natural silencers of these signaling pathways (DUSP6), the multi-component inflammatory cascade underlying MS may be halted (2, 9, 210), overall reducing glia cells and astrocyte activation and inducing myelin damage repair to ultimately restore neural functions.

## Concluding Remarks

Although gaps remain in our detailed understanding of the etiology of MS, the role of physiological, hormonal or cytokine-induced stress conditions triggering reactivation of persistent viral infections and driving aberrant innate and adaptive antiviral immune responses in MS deserves further attention. There is increasing evidence that an inability to adequately control reactivating infection with EBV, pHERV-W and HHV-6 in or near the CNS contributes to the immunopathology in MS, with MHC-II and antigenic mimicry enhancing the autoimmune component of MS pathogenesis. Additional investigations will help understand the conundrum of environmental triggers, reactivating viruses and genetic susceptibility factors in MS.

## Author Contributions

Draft and revision of the manuscript for content: RC, U-CM, JM, RR, and AK. Figure: JM; Table: RR, U-CM, and JM. All authors contributed to the article and approved the submitted version.

## Conflict of Interest

RR is a scientific advisor to IDFISH Technology Inc.

The remaining authors declare that the research was conducted in the absence of any commercial or financial relationships that could be construed as a potential conflict of interest.

## Publisher’s Note

All claims expressed in this article are solely those of the authors and do not necessarily represent those of their affiliated organizations, or those of the publisher, the editors and the reviewers. Any product that may be evaluated in this article, or claim that may be made by its manufacturer, is not guaranteed or endorsed by the publisher.

## Abbreviations

ANO, Anoctamin; BBB, blood-brain-barrier; BCR, B-cell receptor; BTK, Bruton tyrosine kinase; CMV, human cytomegalovirus; CNS, central nervous system; CRYAb, α-B crystallin; CSF, cerebrospinal fluid; CTL, cytotoxic T-lymphocytes; EAE, experimental autoimmune encephalitis EBV, Epstein-Barr Virus; EBER, EBV encoded small RNA; EBNA, Epstein-Barr nuclear antigen; HIV, human immunodeficiency virus; HERV, human endogenous retrovirus; HHV-6, human Herpesvirus-6; HLA, human leukocyte antigen; HSV, Herpes Simplex Virus; IM, infectious mononucleosis; IFN, Interferon; LMP, Latent membrane protein; MAPK, mitogen-activated protein kinase; MBP, myelin basic protein; MOG, myelin oligodendrocyte glycoprotein; MS, Multiple sclerosis; NFL, neurofilament light chain; PLP, Proteolipid protein; PPMS, primary progressive MS; RRMS, relapsing-remitting MS; SPMS, secondary progressive MS; RASGRP, RAS guanyl nucleotide-releasing protein; TCR, T-cell receptor.

## References

[1] Reich DS Lucchinetti CF Calabresi PA. Multiple Sclerosis. N Engl J Med (2018) 378(2):169–80. doi: 10.1056/NEJMra1401483 PMC6942519 29320652

[2] Absinta M Maric D Gharagozloo M Garton T Smith MD Jin J. A Lymphocyte–Microglia–Astrocyte Axis in Chronic Active Multiple Sclerosis. Nature (2021) 597(7878):709–14. doi: 10.1038/s41586-021-03892-7 PMC8719282 34497421

[3] Thompson AJ Banwell BL Barkhof F Carroll WM Coetzee T Comi G. Diagnosis of Multiple Sclerosis: 2017 Revisions of the McDonald Criteria. Lancet Neurol (2018) 17(2):162–73. doi: 10.1016/s1474-4422(17)30470-2 29275977

[4] Filippi M Preziosa P Banwell BL Barkhof F Ciccarelli O De Stefano N. Assessment of Lesions on Magnetic Resonance Imaging in Multiple Sclerosis: Practical Guidelines. Brain (2019) 142(7):1858–75. doi: 10.1093/brain/awz144 PMC6598631 31209474

[5] Chen BY Ghezzi C Villegas B Quon A Radu CG Witte ON. (18)F-FAC PET Visualizes Brain-Infiltrating Leukocytes in a Mouse Model of Multiple Sclerosis. J Nucl Med (2020) 61(5):757–63. doi: 10.2967/jnumed.119.229351 PMC7198381 31653711

[6] Belbasis L Bellou V Evangelou E Ioannidis JP Tzoulaki I. Environmental Risk Factors and Multiple Sclerosis: An Umbrella Review of Systematic Reviews and Meta-Analyses. Lancet Neurol (2015) 14(3):263–73. doi: 10.1016/s1474-4422(14)70267-4 25662901

[7] Ramagopalan SV Dobson R Meier UC Giovannoni G. Multiple Sclerosis: Risk Factors, Prodromes, and Potential Causal Pathways. Lancet Neurol (2010) 9(7):727–39. doi: 10.1016/S1474-4422(10)70094-6 20610348

[8] Bar-Or A Pender MP Khanna R Steinman L Hartung HP Maniar T. Epstein-Barr Virus in Multiple Sclerosis: Theory and Emerging Immunotherapies. Trends Mol Med (2020) 26(3):296–310. doi: 10.1016/j.molmed.2019.11.003 31862243 PMC7106557

[9] Ten Bosch GJ Bolk J Hart B Laman JD. Multiple Sclerosis Is Linked to MAPK ERK Overactivity in Microglia. J Mol Med (Berlin) (2021) 99(8):1033–42. doi: 10.1007/s00109-021-02080-4 PMC8313465 33948692

[10] Tarlinton RE Martynova E Rizvanov AA. Role of Viruses in the Pathogenesis of Multiple Sclerosis. Viruses (2020) 12(6):643. doi: 10.3390/v12060643 PMC7354629 32545816

[11] Guan Y Jakimovski D Ramanathan M Weinstock-Guttman B Zivadinov R. The Role of Epstein-Barr Virus in Multiple Sclerosis: From Molecular Pathophysiology to *In Vivo* Imaging. Neural Regener Res (2019) 14(3):373–86. doi: 10.4103/1673-5374.245462 PMC6334604 30539801

[12] Balfour HH Jr. Odumade OA Schmeling DO Mullan BD Ed JA Knight JA. Behavioral, Virologic, and Immunologic Factors Associated With Acquisition and Severity of Primary Epstein-Barr Virus Infection in University Students. J Infect Dis (2013) 207(1):80–8. doi: 10.1093/infdis/jis646 PMC3523797 23100562

[13] Hadinoto V Shapiro M Sun CC Thorley-Lawson DA. The Dynamics of EBV Shedding Implicate a Central Role for Epithelial Cells in Amplifying Viral Output. PLoS Pathog (2009) 5(7):e1000496. doi: 10.1371/journal.ppat.1000496 19578433 PMC2698984

[14] Price AM Luftig MA. Dynamic Epstein-Barr Virus Gene Expression on the Path to B-Cell Transformation. Adv Virus Res (2014) 88:279–313. doi: 10.1016/b978-0-12-800098-4.00006-4 24373315 PMC4911173

[15] Laichalk LL Hochberg D Babcock GJ Freeman RB Thorley-Lawson DA. The Dispersal of Mucosal Memory B Cells: Evidence From Persistent EBV Infection. Immunity (2002) 16(5):745–54. doi: 10.1016/s1074-7613(02)00318-7 12049725

[16] Maghzi A-H Marta M Bosca I Savoj M-R Etemadifar M Giovannoni G. Epstein–Barr Virus and Multiple Sclerosis. Neuroinflamm: Elsevier (2011) 25–37. doi: 10.1016/b978-0-12-384913-7.00002-2

[17] Thorley-Lawson DA. Epstein-Barr Virus: Exploiting the Immune System. Nat Rev Immunol (2001) 1(1):75–82. doi: 10.1038/35095584 11905817

[18] Shannon-Lowe C Rickinson A. The Global Landscape of EBV-Associated Tumors. Front Oncol (2019) 9:713. doi: 10.3389/fonc.2019.00713 31448229 PMC6691157

[19] Häusler M Ramaekers VT Doenges M Schweizer K Ritter K Schaade L. Neurological Complications of Acute and Persistent Epstein-Barr Virus Infection in Paediatric Patients. J Med Virol (2002) 68(2):253–63. doi: 10.1002/jmv.10201 12210416

[20] Okuno Y Murata T Sato Y. Defective Epstein-Barr Virus in Chronic Active Infection and Haematological Malignancy. Nat Microbiol (2019) 4(3):404–13. doi: 10.1038/s41564-018-0334-0 30664667

[21] Tempera I Lieberman PM. Epigenetic Regulation of EBV Persistence and Oncogenesis. Semin Cancer Biol (2014) 26:22–9. doi: 10.1016/j.semcancer.2014.01.003 PMC4048758 24468737

[22] Middeldorp JM Brink AA van den Brule AJ Meijer CJ. Pathogenic Roles for Epstein-Barr Virus (EBV) Gene Products in EBV-Associated Proliferative Disorders. Crit Rev Oncol Hematol (2003) 45(1):1–36. doi: 10.1016/s1040-8428(02)00078-1 12482570

[23] Skalsky RL Cullen BR. EBV Noncoding RNAs. Curr Top Microbiol Immunol (2015) 391:181–217. doi: 10.1007/978-3-319-22834-1_6 26428375 PMC5685189

[24] Hislop AD Taylor GS Sauce D Rickinson AB. Cellular Responses to Viral Infection in Humans: Lessons From Epstein-Barr Virus. Annu Rev Immunol (2007) 25:587–617. doi: 10.1146/annurev.immunol.25.022106.141553 17378764

[25] Thorley-Lawson DA. EBV Persistence–Introducing the Virus. Curr Top Microbiol Immunol (2015) 390(Pt 1):151–209. doi: 10.1007/978-3-319-22822-8_8 26424647 PMC5125397

[26] Hochberg D Middeldorp JM Catalina M Sullivan JL Luzuriaga K Thorley-Lawson DA. Demonstration of the Burkitt's Lymphoma Epstein-Barr Virus Phenotype in Dividing Latently Infected Memory Cells *In Vivo* . Proc Natl Acad Sci U S A (2004) 101(1):239–44. doi: 10.1073/pnas.2237267100 PMC314169 14688409

[27] Murata T. Encyclopedia of EBV-Encoded Lytic Genes: An Update. Adv Exp Med Biol (2018) 1045:395–412. doi: 10.1007/978-981-10-7230-7_18 29896677

[28] Iwakiri D. Epstein-Barr Virus-Encoded RNAs: Key Molecules in Viral Pathogenesis. Cancers (Basel) (2014) 6(3):1615–30. doi: 10.3390/cancers6031615 PMC4190559 25101570

[29] Qiu J Cosmopoulos K Pegtel M Hopmans E Murray P Middeldorp J. A Novel Persistence Associated EBV miRNA Expression Profile is Disrupted in Neoplasia. PLoS Pathog (2011) 7(8):e1002193. doi: 10.1371/journal.ppat.1002193 21901094 PMC3161978

[30] Iizasa H Kim H Kartika AV Kanehiro Y Yoshiyama H. Role of Viral and Host microRNAs in Immune Regulation of Epstein-Barr Virus-Associated Diseases. Front Immunol (2020) 11:367. doi: 10.3389/fimmu.2020.00367 32194570 PMC7062708

[31] Baglio SR van Eijndhoven MA Koppers-Lalic D Berenguer J Lougheed SM Gibbs S. Sensing of Latent EBV Infection Through Exosomal Transfer of 5'ppprna. Proc Natl Acad Sci U S A (2016) 113(5):E587–96. doi: 10.1073/pnas.1518130113 PMC4747727 26768848

[32] Dobson R Kuhle J Middeldorp J Giovannoni G. Epstein-Barr-Negative MS: A True Phenomenon? Neurol Neuroimmunol Neuroinflamm (2017) 4(2):e318. doi: 10.1212/nxi.0000000000000318 28203615 PMC5292929

[33] Abrahamyan S Eberspächer B Hoshi MM Aly L Luessi F Groppa S. Complete Epstein-Barr Virus Seropositivity in a Large Cohort of Patients With Early Multiple Sclerosis. J Neurol Neurosurg Psychiatry (2020) 91(7):681–6. doi: 10.1136/jnnp-2020-322941 PMC7361012 32371533

[34] Jafari N van Nierop GP Verjans GM Osterhaus AD Middeldorp JM Hintzen RQ. No Evidence for Intrathecal IgG Synthesis to Epstein Barr Virus Nuclear Antigen-1 in Multiple Sclerosis. J Clin Virol (2010) 49(1):26–31. doi: 10.1016/j.jcv.2010.06.007 20638898

[35] Handel AE Williamson AJ Disanto G Handunnetthi L Giovannoni G Ramagopalan SV. An Updated Meta-Analysis of Risk of Multiple Sclerosis Following Infectious Mononucleosis. PLoS One (2010) 5(9):e12496. doi: 10.1371/journal.pone.0012496 20824132 PMC2931696

[36] Thacker EL Mirzaei F Ascherio A. Infectious Mononucleosis and Risk for Multiple Sclerosis: A Meta-Analysis. Ann Neurol (2006) 59(3):499–503. doi: 10.1002/ana.20820 16502434

[37] Disanto G Hall C Lucas R Ponsonby AL Berlanga-Taylor AJ Giovannoni G. Assessing Interactions Between HLA-DRB1*15 and Infectious Mononucleosis on the Risk of Multiple Sclerosis. Mult Scler (2013) 19(10):1355–8. doi: 10.1177/1352458513477231 23413297

[38] Munger KL Levin LI O'Reilly EJ Falk KI Ascherio A. Anti-Epstein-Barr Virus Antibodies as Serological Markers of Multiple Sclerosis: A Prospective Study Among United States Military Personnel. Mult Scler (2011) 17(10):1185–93. doi: 10.1177/1352458511408991 PMC3179777 21685232

[39] Farrell RA Antony D Wall GR Clark DA Fisniku L Swanton J. Humoral Immune Response to EBV in Multiple Sclerosis Is Associated With Disease Activity on MRI. Neurology (2009) 73(1):32–8. doi: 10.1212/WNL.0b013e3181aa29fe PMC2848585 19458321

[40] Lunemann JD Tintore M Messmer B Strowig T Rovira A Perkal H. Elevated Epstein-Barr Virus-Encoded Nuclear Antigen-1 Immune Responses Predict Conversion to Multiple Sclerosis. Ann Neurol (2010) 67(2):159–69. doi: 10.1002/ana.21886 PMC2848293 20225269

[41] Ascherio A Munger KL. Environmental Risk Factors for Multiple Sclerosis. Part I: The Role of Infection. Ann Neurol (2007) 61(4):288–99. doi: 10.1002/ana.21117 17444504

[42] Sisay S Lopez-Lozano L Mickunas M Quiroga-Fernandez A Palace J Warnes G. Untreated Relapsing Remitting Multiple Sclerosis Patients Show Antibody Production Against Latent Epstein Barr Virus (EBV) Antigens Mainly in the Periphery and Innate Immune IL-8 Responses Preferentially in the CNS. J Neuroimmunol (2017) 306:40–5. doi: 10.1016/j.jneuroim.2017.02.017 28385186

[43] Chen MR Middeldorp JM Hayward SD. Separation of the Complex DNA Binding Domain of EBNA-1 Into DNA Recognition and Dimerization Subdomains of Novel Structure. J Virol (1993) 67(8):4875–85. doi: 10.1128/jvi.67.8.4875-4885.1993 PMC237875 8392621

[44] Middeldorp JM. Epstein-Barr Virus-Specific Humoral Immune Responses in Health and Disease. Curr Top Microbiol Immunol (2015) 391:289–323. doi: 10.1007/978-3-319-22834-1_10 26428379

[45] Wang Z Kennedy PG Dupree C Wang M Lee C Pointon T. Antibodies From Multiple Sclerosis Brain Identified Epstein-Barr Virus Nuclear Antigen 1 & 2 Epitopes Which Are Recognized by Oligoclonal Bands. J Neuroimmune Pharmacol (2021) 16(3):567–80. doi: 10.1007/s11481-020-09948-1 PMC7431217 32808238

[46] Hecker M Fitzner B Wendt M Lorenz P Flechtner K Steinbeck F. High-Density Peptide Microarray Analysis of IgG Autoantibody Reactivities in Serum and Cerebrospinal Fluid of Multiple Sclerosis Patients. Mol Cell Proteomics (2016) 15(4):1360–80. doi: 10.1074/mcp.M115.051664 PMC4824861 26831522

[47] Capone G Calabrò M Lucchese G Fasano C Girardi B Polimeno L. Peptide Matching Between Epstein-Barr Virus and Human Proteins. Pathog Dis (2013) 69(3):205–12. doi: 10.1111/2049-632x.12066 23873730

[48] Leen A Meij P Redchenko I Middeldorp J Bloemena E Rickinson A. Differential Immunogenicity of Epstein-Barr Virus Latent-Cycle Proteins for Human CD4(+) T-Helper 1 Responses. J Virol (2001) 75(18):8649–59. doi: 10.1128/jvi.75.18.8649-8659.2001 PMC115110 11507210

[49] Meij P Leen A Rickinson AB Verkoeijen S Vervoort MB Bloemena E. Identification and Prevalence of CD8(+) T-Cell Responses Directed Against Epstein-Barr Virus-Encoded Latent Membrane Protein 1 and Latent Membrane Protein 2. Int J Cancer (2002) 99(1):93–9. doi: 10.1002/ijc.10309 11948498

[50] Godbout JP Glaser R. Stress-Induced Immune Dysregulation: Implications for Wound Healing, Infectious Disease and Cancer. J Neuroimmune Pharmacol (2006) 1(4):421–7. doi: 10.1007/s11481-006-9036-0 18040814

[51] Miller AH Raison CL. The Role of Inflammation in Depression: From Evolutionary Imperative to Modern Treatment Target. Nat Rev Immunol (2016) 16(1):22–34. doi: 10.1038/nri.2015.5 26711676 PMC5542678

[52] Casiraghi C Dorovini-Zis K Horwitz MS. Epstein-Barr Virus Infection of Human Brain Microvessel Endothelial Cells: A Novel Role in Multiple Sclerosis. J Neuroimmunol (2011) 230(1-2):173–7. doi: 10.1016/j.jneuroim.2010.08.003 20826008

[53] Larochelle C Wasser B Jamann H Löffel JT Cui Q-L Tastet O. Pro-Inflammatory T Helper 17 Directly Harms Oligodendrocytes in Neuroinflammation. Proc Natl Acad Sci (2021) 118(34):e2025813118. doi: 10.1073/pnas.2025813118 34417310 PMC8403833

[54] Salloum N Hussein HM Jammaz R Jiche S Uthman IW Abdelnoor AM. Epstein-Barr Virus DNA Modulates Regulatory T-Cell Programming in Addition to Enhancing Interleukin-17A Production *via* Toll-Like Receptor 9. PLoS One (2018) 13(7):e0200546. doi: 10.1371/journal.pone.0200546 29995930 PMC6040775

[55] Damania B Münz C. Immunodeficiencies That Predispose to Pathologies by Human Oncogenic γ-Herpesviruses. FEMS Microbiol Rev (2019) 43(2):181–92. doi: 10.1093/femsre/fuy044 PMC6435449 30649299

[56] Pender MP Burrows SR. Epstein-Barr Virus and Multiple Sclerosis: Potential Opportunities for Immunotherapy. Clin Transl Immunol (2014) 3(10):e27. doi: 10.1038/cti.2014.25 PMC4237030 25505955

[57] Pender MP Csurhes PA Burrows JM Burrows SR. Defective T-Cell Control of Epstein-Barr Virus Infection in Multiple Sclerosis. Clin Transl Immunol (2017) 6(1):e126. doi: 10.1038/cti.2016.87 PMC5292561 28197337

[58] Venken K Hellings N Liblau R Stinissen P. Disturbed Regulatory T Cell Homeostasis in Multiple Sclerosis. Trends Mol Med (2010) 16(2):58–68. doi: 10.1016/j.molmed.2009.12.003 20159585

[59] van Langelaar J Rijvers L Smolders J van Luijn MM. B and T Cells Driving Multiple Sclerosis: Identity, Mechanisms and Potential Triggers. Front Immunol (2020) 11:760. doi: 10.3389/fimmu.2020.00760 32457742 PMC7225320

[60] Schafflick D Xu CA. Integrated Single Cell Analysis of Blood and Cerebrospinal Fluid Leukocytes in Multiple Sclerosis. Nat Commun (2020) 11(1):247. doi: 10.1038/s41467-019-14118-w 31937773 PMC6959356

[61] Serafini B Rosicarelli B Veroni C Mazzola GA Aloisi F. Epstein-Barr Virus-Specific CD8 T Cells Selectively Infiltrate the Brain in Multiple Sclerosis and Interact Locally With Virus-Infected Cells: Clue for a Virus-Driven Immunopathological Mechanism. J Virol (2019) 93(24):e00980–19. doi: 10.1128/jvi.00980-19 PMC6880158 31578295

[62] Lossius A Johansen JN Vartdal F Robins H Jūratė Šaltytė B Holmøy T. High-Throughput Sequencing of TCR Repertoires in Multiple Sclerosis Reveals Intrathecal Enrichment of EBV-Reactive CD8+ T Cells. Eur J Immunol (2014) 44(11):3439–52. doi: 10.1002/eji.201444662 25103993

[63] van Nierop GP Mautner J Mitterreiter JG Hintzen RQ Verjans GM. Intrathecal CD8 T-Cells of Multiple Sclerosis Patients Recognize Lytic Epstein-Barr Virus Proteins. Mult Scler (2016) 22(3):279–91. doi: 10.1177/1352458515588581 26041797

[64] Fransen NL Hsiao CC van der Poel M Engelenburg HJ Verdaasdonk K Vincenten MCJ. Tissue-Resident Memory T Cells Invade the Brain Parenchyma in Multiple Sclerosis White Matter Lesions. Brain (2020) 143(6):1714–30. doi: 10.1093/brain/awaa117 32400866

[65] Jelcic I Al Nimer F Wang J Lentsch V Planas R Jelcic I. Memory B Cells Activate Brain-Homing, Autoreactive CD4(+) T Cells in Multiple Sclerosis. Cell (2018) 175(1):85–100.e23. doi: 10.1016/j.cell.2018.08.011 30173916 PMC6191934

[66] Pender MP. The Essential Role of Epstein-Barr Virus in the Pathogenesis of Multiple Sclerosis. Neuroscientist (2011) 17(4):351–67. doi: 10.1177/1073858410381531 PMC3764840 21075971

[67] Minagar A Alexander JS. Blood-Brain Barrier Disruption in Multiple Sclerosis. Mult Scler (2003) 9(6):540–9. doi: 10.1191/1352458503ms965oa 14664465

[68] Ortiz GG Pacheco-Moisés FP Macías-Islas M Flores-Alvarado LJ Mireles-Ramírez MA González-Renovato ED. Role of the Blood-Brain Barrier in Multiple Sclerosis. Arch Med Res (2014) 45(8):687–97. doi: 10.1016/j.arcmed.2014.11.013 25431839

[69] Horng S Therattil A Moyon S Gordon A Kim K Argaw AT. Astrocytic Tight Junctions Control Inflammatory CNS Lesion Pathogenesis. J Clin Invest (2017) 127(8):3136–51. doi: 10.1172/jci91301 PMC5531407 28737509

[70] Ponath G Park C Pitt D. The Role of Astrocytes in Multiple Sclerosis. Front Immunol (2018) 9:217. doi: 10.3389/fimmu.2018.00217 29515568 PMC5826071

[71] Dong Y Yong VW. When Encephalitogenic T Cells Collaborate With Microglia in Multiple Sclerosis. Nat Rev Neurol (2019) 15(12):704–17. doi: 10.1038/s41582-019-0253-6 31527807

[72] Kunkl M Frascolla S Amormino C Volpe E. Tuosto L. T Helper Cells: The Modulators of Inflammation in Multiple Sclerosis. Cells (2020) 9(2):482. doi: 10.3390/cells9020482 PMC7072830 32093011

[73] Jilek S Schluep M Meylan P Vingerhoets F Guignard L Monney A. Strong EBV-Specific CD8+ T-Cell Response in Patients With Early Multiple Sclerosis. Brain (2008) 131(Pt 7):1712–21. doi: 10.1093/brain/awn108 18550621

[74] Angelini DF Serafini B Piras E Severa M Coccia EM Rosicarelli B. Increased CD8+ T Cell Response to Epstein-Barr Virus Lytic Antigens in the Active Phase of Multiple Sclerosis. PLoS Pathog (2013) 9(4):e1003220. doi: 10.1371/journal.ppat.1003220 23592979 PMC3623710

[75] Lünemann JD Edwards N Muraro PA Hayashi S Cohen JI Münz C. Increased Frequency and Broadened Specificity of Latent EBV Nuclear Antigen-1-Specific T Cells in Multiple Sclerosis. Brain (2006) 129(Pt 6):1493–506. doi: 10.1093/brain/awl067 16569670

[76] Geginat J Paroni M Pagani M Galimberti D De Francesco R Scarpini E. The Enigmatic Role of Viruses in Multiple Sclerosis: Molecular Mimicry or Disturbed Immune Surveillance? Trends Immunol (2017) 38(7):498–512. doi: 10.1016/j.it.2017.04.006 28549714 PMC7185415

[77] Soldan SS Su C Lamontagne RJ Grams N Lu F Zhang Y. Epigenetic Plasticity Enables CNS-Trafficking of EBV-Infected B Lymphocytes. PLoS Pathog (2021) 17(6):e1009618. doi: 10.1371/journal.ppat.1009618 34106998 PMC8216538

[78] Zomer A Vendrig T Hopmans ES van Eijndhoven M Middeldorp JM Pegtel DM. Exosomes: Fit to Deliver Small RNA. Commun Integr Biol (2010) 3(5):447–50. doi: 10.4161/cib.3.5.12339 PMC2974077 21057637

[79] Pegtel DM Peferoen L Amor S. Extracellular Vesicles as Modulators of Cell-to-Cell Communication in the Healthy and Diseased Brain. Philos Trans R Soc Lond B Biol Sci (2014) 369(1652):20130516. doi: 10.1098/rstb.2013.0516 25135977 PMC4142037

[80] Lassmann H Niedobitek G Aloisi F Middeldorp JM. Epstein-Barr Virus in the Multiple Sclerosis Brain: A Controversial Issue–Report on a Focused Workshop Held in the Centre for Brain Research of the Medical University of Vienna, Austria. Brain (2011) 134(Pt 9):2772–86. doi: 10.1093/brain/awr197 PMC3170536 21846731

[81] Morré SA van Beek J De Groot CJ Killestein J Meijer CJ Polman CH. Is Epstein-Barr Virus Present in the CNS of Patients With MS? Neurology (2001) 56(5):692. doi: 10.1212/wnl.56.5.692 11245733

[82] Serafini B Rosicarelli B Franciotta D Magliozzi R Reynolds R Cinque P. Dysregulated Epstein-Barr Virus Infection in the Multiple Sclerosis Brain. J Exp Med (2007) 204(12):2899–912. doi: 10.1084/jem.20071030 PMC2118531 17984305

[83] Peferoen LA Lamers F Lodder LN Gerritsen WH Huitinga I Melief J. Epstein Barr Virus is Not a Characteristic Feature in the Central Nervous System in Established Multiple Sclerosis. Brain (2010) 133(Pt 5):e137. doi: 10.1093/brain/awp296 19917644

[84] Willis SN Stadelmann C Rodig SJ Caron T Gattenloehner S Mallozzi SS. Epstein-Barr Virus Infection Is Not a Characteristic Feature of Multiple Sclerosis Brain. Brain (2009) 132(Pt 12):3318–28. doi: 10.1093/brain/awp200 PMC2792367 19638446

[85] Sargsyan SA Shearer AJ Ritchie AM Burgoon MP Anderson S Hemmer B. Absence of Epstein-Barr Virus in the Brain and CSF of Patients With Multiple Sclerosis. Neurology (2010) 74(14):1127–35. doi: 10.1212/WNL.0b013e3181d865a1 PMC2865779 20220124

[86] Tzartos JS Khan G Vossenkamper A Cruz-Sadaba M Lonardi S Sefia E. Association of Innate Immune Activation With Latent Epstein-Barr Virus in Active MS Lesions. Neurology (2012) 78(1):15–23. doi: 10.1212/WNL.0b013e31823ed057 22156987

[87] Moreno MA Or-Geva N Aftab BT Khanna R Croze E Steinman L. Molecular Signature of Epstein-Barr Virus Infection in MS Brain Lesions. Neurol Neuroimmunol Neuroinflamm (2018) 5(4):e466. doi: 10.1212/nxi.0000000000000466 29892607 PMC5994704

[88] Hassani A Corboy JR Al-Salam S Khan G. Epstein-Barr Virus is Present in the Brain of Most Cases of Multiple Sclerosis and may Engage More Than Just B Cells. PLoS One (2018) 13(2):e0192109. doi: 10.1371/journal.pone.0192109 29394264 PMC5796799

[89] Veroni C Serafini B Rosicarelli B Fagnani C Aloisi F. Transcriptional Profile and Epstein-Barr Virus Infection Status of Laser-Cut Immune Infiltrates From the Brain of Patients With Progressive Multiple Sclerosis. J Neuroinflammation (2018) 15(1):18. doi: 10.1186/s12974-017-1049-5 29338732 PMC5771146

[90] Vomhof-DeKrey EE Yates J Hägglöf T Lanthier P Amiel E Veerapen N. Cognate Interaction With iNKT Cells Expands IL-10-Producing B Regulatory Cells. Proc Natl Acad Sci U S A (2015) 112(40):12474–9. doi: 10.1073/pnas.1504790112 PMC4603516 26392556

[91] Rustenhoven J Drieu A Mamuladze T de Lima KA Dykstra T Wall M. Functional Characterization of the Dural Sinuses as a Neuroimmune Interface. Cell (2021) 184(4):1000–16.e27. doi: 10.1016/j.cell.2020.12.040 33508229 PMC8487654

[92] Stern JN Yaari G Vander Heiden JA Church G Donahue WF Hintzen RQ. B Cells Populating the Multiple Sclerosis Brain Mature in the Draining Cervical Lymph Nodes. Sci Transl Med (2014) 6(248):248ra107. doi: 10.1126/scitranslmed.3008879 PMC4388137 25100741

[93] Serafini B Rosicarelli B Aloisi F Stigliano E. Epstein-Barr Virus in the Central Nervous System and Cervical Lymph Node of a Patient With Primary Progressive Multiple Sclerosis. J Neuropathol Exp Neurol (2014) 73(7):729–31. doi: 10.1097/nen.0000000000000082 24918642

[94] Ebringer A. Ankylosing Spondylitis, Immune-Response-Genes and Molecular Mimicry. Lancet (1979) 1(8127):1186. doi: 10.1016/s0140-6736(79)91861-0 86900

[95] Ramasamy R Joseph B Whittall T. Potential Molecular Mimicry Between the Human Endogenous Retrovirus W Family Envelope Proteins and Myelin Proteins in Multiple Sclerosis. Immunol Lett (2017) 183:79–85. doi: 10.1016/j.imlet.2017.02.003 28189601

[96] Kakalacheva K Munz C Lunemann JD. Viral Triggers of Multiple Sclerosis. Biochim Biophys Acta (2011) 1812(2):132–40. doi: 10.1016/j.bbadis.2010.06.012 PMC7126972 20600868

[97] Chastain EM Miller SD. Molecular Mimicry as an Inducing Trigger for CNS Autoimmune Demyelinating Disease. Immunol Rev (2012) 245(1):227–38. doi: 10.1111/j.1600-065X.2011.01076.x PMC3586283 22168423

[98] Wucherpfennig KW Strominger JL. Molecular Mimicry in T Cell-Mediated Autoimmunity: Viral Peptides Activate Human T Cell Clones Specific for Myelin Basic Protein. Cell (1995) 80(5):695–705. doi: 10.1016/0092-8674(95)90348-8 7534214 PMC7133435

[99] Lang HL Jacobsen H Ikemizu S Andersson C Harlos K Madsen L. A Functional and Structural Basis for TCR Cross-Reactivity in Multiple Sclerosis. Nat Immunol (2002) 3(10):940–3. doi: 10.1038/ni835 12244309

[100] Zdimerova H Murer A Engelmann C Raykova A Deng Y Gujer C. Attenuated Immune Control of Epstein–Barr Virus in Humanized Mice Is Associated With the Multiple Sclerosis Risk Factor HLA-Dr15. Eur J Immunol (2021) 51(1):64–75. doi: 10.1002/eji.202048655 32949466

[101] Lünemann JD Jelcić I Roberts S Lutterotti A Tackenberg B Martin R. EBNA1-Specific T Cells From Patients With Multiple Sclerosis Cross React With Myelin Antigens and Co-Produce IFN-Gamma and IL-2. J Exp Med (2008) 205(8):1763–73. doi: 10.1084/jem.20072397 PMC2525578 18663124

[102] Ramasamy R Mohammed F Meier UC. HLA DR2b-Binding Peptides From Human Endogenous Retrovirus Envelope, Epstein-Barr Virus and Brain Proteins in the Context of Molecular Mimicry in Multiple Sclerosis. Immunol Lett (2020) 217:15–24. doi: 10.1016/j.imlet.2019.10.017 31689443

[103] Jog NR McClain MT Heinlen LD Gross T Towner R Guthridge JM. Epstein Barr Virus Nuclear Antigen 1 (EBNA-1) Peptides Recognized by Adult Multiple Sclerosis Patient Sera Induce Neurologic Symptoms in a Murine Model. J Autoimmun (2020) 106:102332. doi: 10.1016/j.jaut.2019.102332 31515129 PMC6930324

[104] Tengvall K Huang J Hellstrom C Kammer P Bistrom M Ayoglu B. Molecular Mimicry Between Anoctamin 2 and Epstein-Barr Virus Nuclear Antigen 1 Associates With Multiple Sclerosis Risk. Proc Natl Acad Sci U S A (2019) 116(34):16955–60. doi: 10.1073/pnas.1902623116 PMC6708327 31375628

[105] van Sechel AC Bajramovic JJ van Stipdonk MJ Persoon-Deen C Geutskens SB van Noort JM. EBV-Induced Expression and HLA-DR-Restricted Presentation by Human B Cells of Alpha B-Crystallin, a Candidate Autoantigen in Multiple Sclerosis. J Immunol (1999) 162(1):129–35. 9886378

[106] van Noort JM Bsibsi M Gerritsen WH van der Valk P Bajramovic JJ Steinman L. Alphab-Crystallin Is a Target for Adaptive Immune Responses and a Trigger of Innate Responses in Preactive Multiple Sclerosis Lesions. J Neuropathol Exp Neurol (2010) 69(7):694–703. doi: 10.1097/NEN.0b013e3181e4939c 20535035

[107] Lindsey JW deGannes SL Pate KA Zhao X. Antibodies Specific for Epstein-Barr Virus Nuclear Antigen-1 Cross-React With Human Heterogeneous Nuclear Ribonucleoprotein L. Mol Immunol (2016) 69:7–12. doi: 10.1016/j.molimm.2015.11.007 26637929 PMC4698215

[108] Lindsey JW. Antibodies to the Epstein-Barr Virus Proteins BFRF3 and BRRF2 Cross-React With Human Proteins. J Neuroimmunol (2017) 310:131–4. doi: 10.1016/j.jneuroim.2017.07.013 28778437

[109] Dooley MM de Gannes SL Fu KA Lindsey JW. The Increased Antibody Response to Epstein-Barr Virus in Multiple Sclerosis Is Restricted to Selected Virus Proteins. J Neuroimmunol (2016) 299:147–51. doi: 10.1016/j.jneuroim.2016.08.016 27725113

[110] Lomakin Y Arapidi GP Chernov A Ziganshin R Tcyganov E Lyadova I. Exposure to the Epstein-Barr Viral Antigen Latent Membrane Protein 1 Induces Myelin-Reactive Antibodies *In Vivo* . Front Immunol (2017) 8:777. doi: 10.3389/fimmu.2017.00777 28729867 PMC5498468

[111] Woulfe J Gray MT Ganesh MS Middeldorp JM. Human Serum Antibodies Against EBV Latent Membrane Protein 1 Cross-React With α-Synuclein. Neurol Neuroimmunol Neuroinflamm (2016) 3(4):e239. doi: 10.1212/nxi.0000000000000239 27218119 PMC4864620

[112] Kanduc D Shoenfeld Y. From Anti-EBV Immune Responses to the EBV Diseasome *via* Cross-Reactivity. Glob Med Genet (2020) 7(2):51–63. doi: 10.1055/s-0040-1715641 32939516 PMC7490125

[113] Tejada-Simon MV Zang YC Hong J Rivera VM Zhang JZ. Cross-Reactivity With Myelin Basic Protein and Human Herpesvirus-6 in Multiple Sclerosis. Ann Neurol (2003) 53(2):189–97. doi: 10.1002/ana.10425 12557285

[114] Bajramović JJ Plomp AC Goes A Koevoets C Newcombe J Cuzner ML. Presentation of Alpha B-Crystallin to T Cells in Active Multiple Sclerosis Lesions: An Early Event Following Inflammatory Demyelination. J Immunol (2000) 164(8):4359–66. doi: 10.4049/jimmunol.164.8.4359 10754336

[115] Sutkowski N Conrad B Thorley-Lawson DA Huber BT. Epstein-Barr Virus Transactivates the Human Endogenous Retrovirus HERV-K18 That Encodes a Superantigen. Immunity (2001) 15(4):579–89. doi: 10.1016/s1074-7613(01)00210-2 11672540

[116] Hsiao FC Tai AK Deglon A Sutkowski N Longnecker R Huber BT. EBV LMP-2A Employs a Novel Mechanism to Transactivate the HERV-K18 Superantigen Through its ITAM. Virology (2009) 385(1):261–6. doi: 10.1016/j.virol.2008.11.025 19070345

[117] Mameli G Madeddu G Mei A Uleri E Poddighe L Delogu LG. Activation of MSRV-Type Endogenous Retroviruses During Infectious Mononucleosis and Epstein-Barr Virus Latency: The Missing Link With Multiple Sclerosis? PLoS One (2013) 8(11):e78474. doi: 10.1371/journal.pone.0078474 24236019 PMC3827255

[118] Morandi E Tarlinton RE Gran B. Multiple Sclerosis Between Genetics and Infections: Human Endogenous Retroviruses in Monocytes and Macrophages. Front Immunol (2015) 6:647. doi: 10.3389/fimmu.2015.00647 26734011 PMC4689809

[119] Garcia-Montojo M Rodriguez-Martin E Ramos-Mozo P Ortega-Madueño I Dominguez-Mozo MI Arias-Leal A. Syncytin-1/HERV-W Envelope is an Early Activation Marker of Leukocytes and Is Upregulated in Multiple Sclerosis Patients. Eur J Immunol (2020) 50(5):685–94. doi: 10.1002/eji.201948423 32012247

[120] Frey TR Akinyemi IA Burton EM Bhaduri-McIntosh S McIntosh MT. An Ancestral Retrovirus Envelope Protein Regulates Persistent Gammaherpesvirus Lifecycles. Front Microbiol (2021) 12:708404. doi: 10.3389/fmicb.2021.708404 34434177 PMC8381357

[121] Gruchot J Kremer D Küry P. Neural Cell Responses Upon Exposure to Human Endogenous Retroviruses. Front Genet (2019) 10:655. doi: 10.3389/fgene.2019.00655 31354794 PMC6637040

[122] Zhou Y Liu L Liu Y Zhou P Yan Q Yu H. Implication of Human Endogenous Retrovirus W Family Envelope in Hepatocellular Carcinoma Promotes MEK/ERK-Mediated Metastatic Invasiveness and Doxorubicin Resistance. Cell Death Discov (2021) 7(1):177. doi: 10.1038/s41420-021-00562-5 34238921 PMC8266889

[123] Tao C Simpson S Jr Taylor BV van der Mei I. Association Between Human Herpesvirus & Human Endogenous Retrovirus and MS Onset & Progression. J Neurol Sci (2017) 372:239–49. doi: 10.1016/j.jns.2016.11.060 28017222

[124] Gate D Saligrama N Leventhal O Yang AC Unger MS Middeldorp J. Clonally Expanded CD8 T Cells Patrol the Cerebrospinal Fluid in Alzheimer's Disease. Nature (2020) 577(7790):399–404. doi: 10.1038/s41586-019-1895-7 31915375 PMC7445078

[125] Alsema AM Jiang Q Kracht L Gerrits E Dubbelaar ML Miedema A. Profiling Microglia From Alzheimer's Disease Donors and Non-Demented Elderly in Acute Human Postmortem Cortical Tissue. Front Mol Neurosci (2020) 13:134. doi: 10.3389/fnmol.2020.00134 33192286 PMC7655794

[126] Bar-Or A Li R. Cellular Immunology of Relapsing Multiple Sclerosis: Interactions, Checks, and Balances. Lancet Neurol (2021) 20(6):470–83. doi: 10.1016/s1474-4422(21)00063-6 33930317

[127] Clark IC Gutiérrez-Vázquez C Wheeler MA Li Z Rothhammer V Linnerbauer M. Barcoded Viral Tracing of Single-Cell Interactions in Central Nervous System Inflammation. Science (2021) 372(6540):eabf1230. doi: 10.1126/science.abf1230 33888612 PMC8157482

[128] Alvarenga MOP Frazão DR de Matos IG Bittencourt LO Fagundes NCF Rösing CK. Is There Any Association Between Neurodegenerative Diseases and Periodontitis? A Systematic Review. Front Aging Neurosci (2021) 13:651437. doi: 10.3389/fnagi.2021.651437 34108875 PMC8180549

[129] Haase S Wilck N Haghikia A Gold R Mueller DN Linker RA. The Role of the Gut Microbiota and Microbial Metabolites in Neuroinflammation. Eur J Immunol (2020) 50(12):1863–70. doi: 10.1002/eji.201847807 33188704

[130] Koike R Nodomi K Watanabe N Ogata Y Takeichi O Takei M. Butyric Acid in Saliva of Chronic Periodontitis Patients Induces Transcription of the EBV Lytic Switch Activator BZLF1: A Pilot Study. In Vivo (2020) 34(2):587–94. doi: 10.21873/invivo.11811 PMC7157893 32111757

[131] Novalic Z van Rossen T Greijer A Middeldorp J. Agents and Approaches for Lytic Induction Therapy of Epstein-Barr Virus Associated Malignancies. Med Chem (2016) 6:449–66. doi: 10.4172/2161-0444.1000384

[132] Alvarez-Lafuente R De Las Heras V Bartolomé M Picazo JJ Arroyo R. Beta-Interferon Treatment Reduces Human Herpesvirus-6 Viral Load in Multiple Sclerosis Relapses But Not in Remission. Eur Neurol (2004) 52(2):87–91. doi: 10.1159/000079936 15273429

[133] Petersen T Møller-Larsen A Ellermann-Eriksen S Thiel S Christensen T. Effects of Interferon-Beta Therapy on Elements in the Antiviral Immune Response Towards the Human Herpesviruses EBV, HSV, and VZV, and to the Human Endogenous Retroviruses HERV-H and HERV-W in Multiple Sclerosis. J Neuroimmunol (2012) 249(1-2):105–8. doi: 10.1016/j.jneuroim.2012.04.013 22608883

[134] Meier UC Giovannoni G Tzartos JS Khan G. Translational Mini-Review Series on B Cell Subsets in Disease. B Cells in Multiple Sclerosis: Drivers of Disease Pathogenesis and Trojan Horse for Epstein-Barr Virus Entry to the Central Nervous System? Clin Exp Immunol (2012) 167(1):1–6. doi: 10.1111/j.1365-2249.2011.04446.x 22132878 PMC3248080

[135] Mulero P Midaglia L Montalban X. Ocrelizumab: A New Milestone in Multiple Sclerosis Therapy. Ther Adv Neurol Disord (2018) 11:1756286418773025. doi: 10.1177/1756286418773025 29774057 PMC5952271

[136] Gratama JW Oosterveer MA Zwaan FE Lepoutre J Klein G Ernberg I. Eradication of Epstein-Barr Virus by Allogeneic Bone Marrow Transplantation: Implications for Sites of Viral Latency. Proc Natl Acad Sci U S A (1988) 85(22):8693–6. doi: 10.1073/pnas.85.22.8693 PMC282526 2847171

[137] Miller AE Chitnis T Cohen BA Costello K Sicotte NL Stacom R. Autologous Hematopoietic Stem Cell Transplant in Multiple Sclerosis: Recommendations of the National Multiple Sclerosis Society. JAMA Neurol (2021) 78(2):241–6. doi: 10.1001/jamaneurol.2020.4025 33104165

[138] Drosu NC Edelman ER. Tenofovir Prodrugs Potently Inhibit Epstein-Barr Virus Lytic DNA Replication by Targeting the Viral DNA Polymerase. Proc Natl Acad Sci U S A (2020) 117(22):12368–74. doi: 10.1073/pnas.2002392117 PMC7275665 32409608

[139] Maruszak H Brew BJ Giovannoni G Gold J. Could Antiretroviral Drugs be Effective in Multiple Sclerosis? A Case Report. Eur J Neurol (2011) 18(9):e110–1. doi: 10.1111/j.1468-1331.2011.03430.x 21834893

[140] Pender MP Csurhes PA Smith C Douglas NL Neller MA Matthews KK. Epstein-Barr Virus-Specific T Cell Therapy for Progressive Multiple Sclerosis. JCI Insight (2018) 3(22):e124714. doi: 10.1172/jci.insight.124714 PMC6302936 30429369

[141] Ioannides ZA Csurhes PA Douglas NL Mackenroth G Swayne A Thompson KM. Sustained Clinical Improvement in a Subset of Patients With Progressive Multiple Sclerosis Treated With Epstein-Barr Virus-Specific T Cell Therapy. Front Neurol (2021) 12:652811. doi: 10.3389/fneur.2021.652811 33790852 PMC8005645

[142] Marron TU Martinez-Gallo M Yu JE Cunningham-Rundles C. Toll-Like Receptor 4-, 7-, and 8-Activated Myeloid Cells From Patients With X-Linked Agammaglobulinemia Produce Enhanced Inflammatory Cytokines. J Allergy Clin Immunol (2012) 129(1):184–90.e1-4. doi: 10.1016/j.jaci.2011.10.009 22088613 PMC3428022

[143] Menzfeld C John M van Rossum D Regen T Scheffel J Janova H. Tyrphostin AG126 Exerts Neuroprotection in CNS Inflammation by a Dual Mechanism. Glia (2015) 63(6):1083–99. doi: 10.1002/glia.22803 25731696

[144] Incrocci R Barse L Stone A Vagvala S Montesano M Subramaniam V. Epstein-Barr Virus Latent Membrane Protein 2a (LMP2A) Enhances IL-10 Production Through the Activation of Bruton's Tyrosine Kinase and STAT3. Virology (2017) 500:96–102. doi: 10.1016/j.virol.2016.10.015 27792904 PMC5127766

[145] Montalban X Arnold DL Weber MS Staikov I Piasecka-Stryczynska K Willmer J. Placebo-Controlled Trial of an Oral BTK Inhibitor in Multiple Sclerosis. N Engl J Med (2019) 380(25):2406–17. doi: 10.1056/NEJMoa1901981 31075187

[146] Reich DS Arnold DL Vermersch P Bar-Or A Fox RJ Matta A. Safety and Efficacy of Tolebrutinib, an Oral Brain-Penetrant BTK Inhibitor, in Relapsing Multiple Sclerosis: A Phase 2b, Randomised, Double-Blind, Placebo-Controlled Trial. Lancet Neurol (2021) 20(9):729–38. doi: 10.1016/s1474-4422(21)00237-4 PMC8434816 34418400

[147] Bame E Tang H Burns JC Arefayene M Michelsen K Ma B. Next-Generation Bruton's Tyrosine Kinase Inhibitor BIIB091 Selectively and Potently Inhibits B Cell and Fc Receptor Signaling and Downstream Functions in B Cells and Myeloid Cells. Clin Trans Immunol (2021) 10(6):e1295. doi: 10.1002/cti2.1295 PMC8204096 34141433

[148] Rajendran R Böttiger G Dentzien N Rajendran V Sharifi B Ergün S. Effects of FGFR Tyrosine Kinase Inhibition in OLN-93 Oligodendrocytes. Cells (2021) 10(6):1318. doi: 10.3390/cells10061318 34070622 PMC8228431

[149] Suo N Guo YE He B Gu H Xie X. Inhibition of MAPK/ERK Pathway Promotes Oligodendrocytes Generation and Recovery of Demyelinating Diseases. Glia (2019) 67(7):1320–32. doi: 10.1002/glia.23606 PMC6593996 30815939

[150] Zhang Y Li X Ciric B Curtis MT Chen WJ Rostami A. A Dual Effect of Ursolic Acid to the Treatment of Multiple Sclerosis Through Both Immunomodulation and Direct Remyelination. Proc Natl Acad Sci U S A (2020) 117(16):9082–93. doi: 10.1073/pnas.2000208117 PMC7183235 32253301

[151] Soldan SS Anderson EM Frase DM Zhang Y Caruso LB Wang Y. EBNA1 Inhibitors Have Potent and Selective Antitumor Activity in Xenograft Models of Epstein-Barr Virus-Associated. Gastric Cancer (2021) 24(5):1076–88. doi: 10.1007/s10120-021-01193-6 PMC8338878 33929613

[152] Jiang L Lung HL Huang T Lan R Zha S Chan LS. Reactivation of Epstein–Barr Virus by a Dual-Responsive Fluorescent EBNA1-Targeting Agent With Zn2+-Chelating Function. Proc Natl Acad Sci (2019) 116(52):26614–24. doi: 10.1073/pnas.1915372116 PMC6936348 31822610

[153] Höllsberg P Kusk M Bech E Hansen HJ Jakobsen J Haahr S. Presence of Epstein-Barr Virus and Human Herpesvirus 6B DNA in Multiple Sclerosis Patients: Associations With Disease Activity. Acta Neurol Scand (2005) 112(6):395–402. doi: 10.1111/j.1600-0404.2005.00516.x 16281923

[154] Soldan SS Leist TP Juhng KN McFarland HF Jacobson S. Increased Lymphoproliferative Response to Human Herpesvirus Type 6A Variant in Multiple Sclerosis Patients. Ann Neurol (2000) 47(3):306–13. doi: 10.1002/1531-8249(200003)47:3<306::aid-ana5>3.0.co;2-a 10716249

[155] Ortega-Madueño I Garcia-Montojo M Dominguez-Mozo MI Garcia-Martinez A Arias-Leal AM Casanova I. Anti-Human Herpesvirus 6A/B IgG Correlates With Relapses and Progression in Multiple Sclerosis. PLoS One (2014) 9(8):e104836. doi: 10.1371/journal.pone.0104836 25110949 PMC4128748

[156] Hall CB Long CE Schnabel KC Caserta MT McIntyre KM Costanzo MA. Human Herpesvirus-6 Infection in Children. A Prospective Study of Complications and Reactivation. N Engl J Med (1994) 331(7):432–8. doi: 10.1056/nejm199408183310703 8035839

[157] Caserta MT Hall CB Schnabel K McIntyre K Long C Costanzo M. Neuroinvasion and Persistence of Human Herpesvirus 6 in Children. J Infect Dis (1994) 170(6):1586–9. doi: 10.1093/infdis/170.6.1586 7996000

[158] Knox KK Carrigan DR. Active Human Herpesvirus (HHV-6) Infection of the Central Nervous System in Patients With AIDS. J Acquir Immune Defic Syndr Hum Retrovirol (1995) 9(1):69–73. 7712236

[159] Zhang E Bell AJ Wilkie GS Suárez NM Batini C Veal CD. Inherited Chromosomally Integrated Human Herpesvirus 6 Genomes Are Ancient, Intact, and Potentially Able To Reactivate From Telomeres. J Virol (2017) 91(22):e01137–17. doi: 10.1128/jvi.01137-17 PMC5660504 28835501

[160] Leibovitch EC Jacobson S. Evidence Linking HHV-6 With Multiple Sclerosis: An Update. Curr Opin Virol (2014) 9:127–33. doi: 10.1016/j.coviro.2014.09.016 PMC4269240 25462444

[161] Kawano M Seya T Koni I Mabuchi H. Elevated Serum Levels of Soluble Membrane Cofactor Protein (CD46, MCP) in Patients With Systemic Lupus Erythematosus (SLE). Clin Exp Immunol (1999) 116(3):542–6. doi: 10.1046/j.1365-2249.1999.00917.x PMC1905304 10361248

[162] Reynaud JM Jégou JF Welsch JC Horvat B. Human Herpesvirus 6A Infection in CD46 Transgenic Mice: Viral Persistence in the Brain and Increased Production of Proinflammatory Chemokines *via* Toll-Like Receptor 9. J Virol (2014) 88(10):5421–36. doi: 10.1128/jvi.03763-13 PMC4019085 24574405

[163] Leibovitch E Wohler JE Cummings Macri SM Motanic K Harberts E Gaitán MI. Novel Marmoset (Callithrix Jacchus) Model of Human Herpesvirus 6A and 6B Infections: Immunologic, Virologic and Radiologic Characterization. PLoS Pathog (2013) 9(1):e1003138. doi: 10.1371/journal.ppat.1003138 23382677 PMC3561285

[164] Harberts E Yao K Wohler JE Maric D Ohayon J Henkin R. Human Herpesvirus-6 Entry Into the Central Nervous System Through the Olfactory Pathway. Proc Natl Acad Sci U S A (2011) 108(33):13734–9. doi: 10.1073/pnas.1105143108 PMC3158203 21825120

[165] Challoner PB Smith KT Parker JD MacLeod DL Coulter SN Rose TM. Plaque-Associated Expression of Human Herpesvirus 6 in Multiple Sclerosis. Proc Natl Acad Sci U S A (1995) 92(16):7440–4. doi: 10.1073/pnas.92.16.7440 PMC41355 7638210

[166] Goodman AD Mock DJ Powers JM Baker JV Blumberg BM. Human Herpesvirus 6 Genome and Antigen in Acute Multiple Sclerosis Lesions. J Infect Dis (2003) 187(9):1365–76. doi: 10.1086/368172 12717617

[167] Chapenko S Millers A Nora Z Logina I Kukaine R Murovska M. Correlation Between HHV-6 Reactivation and Multiple Sclerosis Disease Activity. J Med Virol (2003) 69(1):111–7. doi: 10.1002/jmv.10258 12436486

[168] Soldan SS Berti R Salem N Secchiero P Flamand L Calabresi PA. Association of Human Herpes Virus 6 (HHV-6) With Multiple Sclerosis: Increased IgM Response to HHV-6 Early Antigen and Detection of Serum HHV-6 DNA. Nat Med (1997) 3(12):1394–7. doi: 10.1038/nm1297-1394 9396611

[169] Tai AK Luka J Ablashi D Huber BT. HHV-6A Infection Induces Expression of HERV-K18-Encoded Superantigen. J Clin Virol (2009) 46(1):47–8. doi: 10.1016/j.jcv.2009.05.019 19505843

[170] Flamand L Stefanescu I Ablashi DV Menezes J. Activation of the Epstein-Barr Virus Replicative Cycle by Human Herpesvirus 6. J Virol (1993) 67(11):6768–77. doi: 10.1128/jvi.67.11.6768-6777.1993 PMC238118 8411380

[171] Flamand L Menezes J. Cyclic AMP-Responsive Element-Dependent Activation of Epstein-Barr Virus Zebra Promoter by Human Herpesvirus 6. J Virol (1996) 70(3):1784–91. doi: 10.1128/JVI.70.3.1784-1791.1996 PMC190004 8627701

[172] Cuomo L Angeloni A Zompetta C Cirone M Calogero A Frati L. Human Herpesvirus 6 Variant A, But Not Variant B, Infects EBV-Positive B Lymphoid Cells, Activating the Latent EBV Genome Through a BZLF-1-Dependent Mechanism. AIDS Res Hum Retroviruses (1995) 11(10):1241–5. doi: 10.1089/aid.1995.11.1241 8573381

[173] Turcanova VL Bundgaard B Hollsberg P. Human Herpesvirus-6B Induces Expression of the Human Endogenous Retrovirus K18-Encoded Superantigen. J Clin Virol (2009) 46(1):15–9. doi: 10.1016/j.jcv.2009.05.015 19505847

[174] Charvet B Reynaud JM Gourru-Lesimple G Perron H Marche PN Horvat B. Induction of Proinflammatory Multiple Sclerosis-Associated Retrovirus Envelope Protein by Human Herpesvirus-6A and CD46 Receptor Engagement. Front Immunol (2018) 9:2803. doi: 10.3389/fimmu.2018.02803 30574140 PMC6291489

[175] Johnson WE. Origins and Evolutionary Consequences of Ancient Endogenous Retroviruses. Nat Rev Microbiol (2019) 17(6):355–70. doi: 10.1038/s41579-019-0189-2 30962577

[176] Lander ES Linton LM Birren B Nusbaum C Zody MC Baldwin J. Initial Sequencing and Analysis of the Human Genome. Nature (2001) 409(6822):860–921. doi: 10.1038/35057062 11237011

[177] Karimi A Esmaili N Ranjkesh M Zolfaghari MA. Expression of Human Endogenous Retroviruses in Pemphigus Vulgaris Patients. Mol Biol Rep (2019) 46(6):6181–6. doi: 10.1007/s11033-019-05053-6 31473891

[178] Morris G Maes M Murdjeva M Puri BK. Do Human Endogenous Retroviruses Contribute to Multiple Sclerosis, and if So How? Mol Neurobiol (2019) 56(4):2590–605. doi: 10.1007/s12035-018-1255-x PMC6459794 30047100

[179] de Parseval N Lazar V Casella JF Benit L Heidmann T. Survey of Human Genes of Retroviral Origin: Identification and Transcriptome of the Genes With Coding Capacity for Complete Envelope Proteins. J Virol (2003) 77(19):10414–22. doi: 10.1128/jvi.77.19.10414-10422.2003 PMC228468 12970426

[180] Mi S Lee X Li X Veldman GM Finnerty H Racie L. Syncytin is a Captive Retroviral Envelope Protein Involved in Human Placental Morphogenesis. Nature (2000) 403(6771):785–9. doi: 10.1038/35001608 10693809

[181] Lokossou AG Toudic C Barbeau B. Implication of Human Endogenous Retrovirus Envelope Proteins in Placental Functions. Viruses (2014) 6(11):4609–27. doi: 10.3390/v6114609 PMC4246240 25421890

[182] Perron H Lalande B Gratacap B Laurent A Genoulaz O Geny C. Isolation of Retrovirus From Patients With Multiple Sclerosis. Lancet (1991) 337(8745):862–3. doi: 10.1016/0140-6736(91)92579-q 1707471

[183] Kremer D Gruchot J Weyers V Oldemeier L Göttle P. pHERV-W Envelope Protein Fuels Microglial Cell-Dependent Damage of Myelinated Axons in Multiple Sclerosis. Proc Natl Acad Sci U S A (2019) 116(30):15216–25. doi: 10.1073/pnas.1901283116 PMC6660731 31213545

[184] Hartung H-P Derfuss T Cree BA Sormani MP Selmaj K Stutters J. Efficacy and Safety of Temelimab in Multiple Sclerosis: Results of a Randomized Phase 2b and Extension Study. Multiple Sclerosis J (2021), 13524585211024997. doi: 10.1177/13524585211024997 34240656

[185] van Horssen J van der Pol S Nijland P Amor S Perron H. Human Endogenous Retrovirus W in Brain Lesions: Rationale for Targeted Therapy in Multiple Sclerosis. Mult Scler Relat Disord (2016) 8:11–8. doi: 10.1016/j.msard.2016.04.006 27456869

[186] Ruprecht K Mayer J. On the Origin of a Pathogenic HERV-W Envelope Protein Present in Multiple Sclerosis Lesions. Proc Natl Acad Sci U S A (2019) 116(40):19791–2. doi: 10.1073/pnas.1911703116 PMC6778242 31455746

[187] International Multiple Sclerosis Genetics C Wellcome Trust Case Control C Sawcer S Hellenthal G Pirinen M Spencer CC. Genetic Risk and a Primary Role for Cell-Mediated Immune Mechanisms in Multiple Sclerosis. Nature (2011) 476(7359):214–9. doi: 10.1038/nature10251 PMC3182531 21833088

[188] Antony JM Deslauriers AM Bhat RK Ellestad KK Power C. Human Endogenous Retroviruses and Multiple Sclerosis: Innocent Bystanders or Disease Determinants? Biochim Biophys Acta (2011) 1812(2):162–76. doi: 10.1016/j.bbadis.2010.07.016 PMC7172332 20696240

[189] Antony JM Ellestad KK Hammond R Imaizumi K Mallet F Warren KG. The Human Endogenous Retrovirus Envelope Glycoprotein, Syncytin-1, Regulates Neuroinflammation and its Receptor Expression in Multiple Sclerosis: A Role for Endoplasmic Reticulum Chaperones in Astrocytes. J Immunol (2007) 179(2):1210–24. doi: 10.4049/jimmunol.179.2.1210 17617614

[190] Chen CP Chen LF Yang SR Chen CY Ko CC Chang GD. Functional Characterization of the Human Placental Fusogenic Membrane Protein Syncytin 2. Biol Reprod (2008) 79(5):815–23. doi: 10.1095/biolreprod.108.069765 18650494

[191] Lokossou AG Toudic C Nguyen PT Elisseeff X Vargas A Rassart É. Endogenous Retrovirus-Encoded Syncytin-2 Contributes to Exosome-Mediated Immunosuppression of T Cells†. Biol Reprod (2020) 102(1):185–98. doi: 10.1093/biolre/ioz124 31318021

[192] Tolosa JM Schjenken JE Clifton VL Vargas A Barbeau B Lowry P. The Endogenous Retroviral Envelope Protein Syncytin-1 Inhibits LPS/PHA-Stimulated Cytokine Responses in Human Blood and Is Sorted Into Placental Exosomes. Placenta (2012) 33(11):933–41. doi: 10.1016/j.placenta.2012.08.004 22999499

[193] Charvet B Pierquin J Brunel J Gorter R Quétard C Horvat B. Human Endogenous Retrovirus Type W Envelope From Multiple Sclerosis Demyelinating Lesions Shows Unique Solubility and Antigenic Characteristics. Virol Sin (2021). doi: 10.1007/s12250-021-00372-0 PMC8558138 33770381

[194] Mameli G Astone V Arru G Marconi S Lovato L Serra C. Brains and Peripheral Blood Mononuclear Cells of Multiple Sclerosis (MS) Patients Hyperexpress MS-Associated Retrovirus/HERV-W Endogenous Retrovirus, But Not Human Herpesvirus 6. J Gen Virol (2007) 88(Pt 1):264–74. doi: 10.1099/vir.0.81890-0 17170460

[195] Dolei A. Endogenous Retroviruses and Human Disease. Expert Rev Clin Immunol (2006) 2(1):149–67. doi: 10.1586/1744666x.2.1.149 20477095

[196] Nissen KK Laska MJ Hansen B Terkelsen T Villesen P Bahrami S. Endogenous Retroviruses and Multiple Sclerosis-New Pieces to the Puzzle. BMC Neurol (2013) 13:111. doi: 10.1186/1471-2377-13-111 23984932 PMC3765820

[197] Arneth B. Up-To-Date Knowledge About the Association Between Multiple Sclerosis and the Reactivation of Human Endogenous Retrovirus Infections. J Neurol (2018) 265(8):1733–9. doi: 10.1007/s00415-018-8783-1 29423611

[198] Arru G Mameli G Astone V Serra C Huang YM Link H. Multiple Sclerosis and HERV-W/MSRV: A Multicentric Study. Int J BioMed Sci (2007) 3(4):292–7. PMC3614662 23675056

[199] Grandi N Tramontano E. HERV Envelope Proteins: Physiological Role and Pathogenic Potential in Cancer and Autoimmunity. Front Microbiol (2018) 9:462. doi: 10.3389/fmicb.2018.00462 29593697 PMC5861771

[200] Firouzi R Rolland A Michel M Jouvin-Marche E Hauw JJ Malcus-Vocanson C. Multiple Sclerosis-Associated Retrovirus Particles Cause T Lymphocyte-Dependent Death With Brain Hemorrhage in Humanized SCID Mice Model. J Neurovirol (2003) 9(1):79–93. doi: 10.1080/13550280390173328 12587071

[201] Perron H Bernard C Bertrand JB Lang AB Popa I Sanhadji K. Endogenous Retroviral Genes, Herpesviruses and Gender in Multiple Sclerosis. J Neurol Sci (2009) 286(1-2):65–72. doi: 10.1016/j.jns.2009.04.034 19447411

[202] Garcia-Montojo M Dominguez-Mozo M Arias-Leal A Garcia-Martinez Á De las Heras V Casanova I. The DNA Copy Number of Human Endogenous Retrovirus-W (MSRV-Type) Is Increased in Multiple Sclerosis Patients and is Influenced by Gender and Disease Severity. PLoS One (2013) 8(1):e53623. doi: 10.1371/journal.pone.0053623 23308264 PMC3538585

[203] Mameli G Serra C Astone V Castellazzi M Poddighe L Fainardi E. Inhibition of Multiple-Sclerosis-Associated Retrovirus as Biomarker of Interferon Therapy. J Neurovirol (2008) 14(1):73–7. doi: 10.1080/13550280701801107 18300077

[204] Porchet H Vidal V Kornmann G Malpass S. Curtin F. A High-Dose Pharmacokinetic Study of a New IgG4 Monoclonal Antibody Temelimab/GNbAC1 Antagonist of an Endogenous Retroviral Protein pHERV-W Env. Clin Ther (2019) 41(9):1737–46. doi: 10.1016/j.clinthera.2019.05.020 31311668

[205] Gold J Marta M Meier UC Christensen T Miller D Altmann D. A Phase II Baseline *Versus* Treatment Study to Determine the Efficacy of Raltegravir (Isentress) in Preventing Progression of Relapsing Remitting Multiple Sclerosis as Determined by Gadolinium-Enhanced MRI: The INSPIRE Study. Mult Scler Relat Disord (2018) 24:123–8. doi: 10.1016/j.msard.2018.06.002 29990894

[206] Zubizarreta I Florez-Grau G Vila G Cabezon R Espana C Andorra M. Immune Tolerance in Multiple Sclerosis and Neuromyelitis Optica With Peptide-Loaded Tolerogenic Dendritic Cells in a Phase 1b Trial. Proc Natl Acad Sci U S A (2019) 116(17):8463–70. doi: 10.1073/pnas.1820039116 PMC6486735 30962374

[207] Saligrama N Zhao F Sikora MJ Serratelli WS Fernandes RA Louis DM. Opposing T Cell Responses in Experimental Autoimmune Encephalomyelitis. Nature (2019) 572(7770):481–7. doi: 10.1038/s41586-019-1467-x PMC7145319 31391585

[208] Krienke C Kolb L Diken E Streuber M Kirchhoff S Bukur T. A Noninflammatory mRNA Vaccine for Treatment of Experimental Autoimmune Encephalomyelitis. Science (2021) 371(6525):145–53. doi: 10.1126/science.aay3638 33414215

[209] Croese T Castellani G Schwartz M. Immune Cell Compartmentalization for Brain Surveillance and Protection. Nat Immunol (2021) 22(9):1083–92. doi: 10.1038/s41590-021-00994-2 34429552

[210] Kumar N Sharma N Mehan S. Connection Between JAK/STAT and Pparγ Signaling During the Progression of Multiple Sclerosis: Insights Into the Modulation of T-Cells and Immune Responses in the Brain. Curr Mol Pharmacol (2021). doi: 10.2174/1874467214666210301121432 33645493

[211] Louveau A Herz J Alme MN Salvador AF Dong MQ Viar KE. CNS Lymphatic Drainage and Neuroinflammation Are Regulated by Meningeal Lymphatic Vasculature. Nat Neurosci (2018) 21(10):1380–91. doi: 10.1038/s41593-018-0227-9 PMC6214619 30224810

